# Prevalence of mental disorders in adult populations from the Global South following exposure to natural hazards: a meta-analysis

**DOI:** 10.1017/S2045796024000672

**Published:** 2024-11-28

**Authors:** A. Kip, S. Valencia, E. Glunz, S. R. Lowe, K.-P. Tam, N. Morina

**Affiliations:** 1 Institute of Psychology, University of Münster, Münster, Germany; 2 Institute of Psychology, Technical University Braunschweig, Braunschweig, Germany; 3 Department of Social and Behavioral Sciences, Yale School of Public Health, New Haven, CT, USA; 4 Division of Social Science, The Hong Kong University of Science and Technology, Hong Kong, China; 5 Department of Psychology, New School for Social Research, New York, NY, USA

**Keywords:** depression, epidemiology, post-traumatic stress disorder, systematic reviews, natural hazards, climate change, meta-analysis

## Abstract

**Aims:**

Although natural hazards (e.g., tropical cyclones, earthquakes) disproportionately affect developing countries, most research on their mental health impact has been conducted in high-income countries. We aimed to summarize prevalences of mental disorders in Global South populations (classified according to the United Nations Human Development Index) affected by natural hazards.

**Methods:**

To identify eligible studies for this meta-analysis, we searched MEDLINE, PsycINFO and Web of Science up to February 13, 2024, for observational studies with a cross-sectional or longitudinal design that reported on at least 100 adult survivors of natural hazards in a Global South population and assessed mental disorders with a validated instrument at least 1 month after onset of the hazard. Main outcomes were the short- and long-term prevalence estimates of mental disorders. The project was registered on the International Prospective Register of Systematic Reviews (CRD42023396622).

**Results:**

We included 77 reports of 75 cross-sectional studies (six included a non-exposed control group) comprising 82,400 individuals. We found high prevalence estimates for post-traumatic stress disorder (PTSD) in the general population (26.0% [95% CI 18.5–36.3]; *I*^2^ = 99.0%) and depression (21.7% [95% CI 10.5–39.6]; *I*^2^ = 99.2%) during the first year following the event, with similar prevalences observed thereafter (i.e., 26.0% and 23.4%, respectively). Results were similar for regions with vs. without recent armed conflict. In displaced samples, the estimated prevalence for PTSD was 46.5% (95% CI 39.0–54.2; *k* = 6; *I*^2^ = 93.3). We furthermore found higher symptom severity in exposed, versus unexposed, individuals. Data on other disorders were scarce, apart from short-term prevalence estimates of generalised anxiety disorder (15.9% [95% CI 4.7–42.0]; *I*^2^ = 99.4).

**Conclusions:**

Global South populations exposed to natural hazards report a substantial burden of mental disease. These findings require further attention and action in terms of implementation of mental health policies and low-threshold interventions in the Global South in the aftermath of natural hazards. However, to accurately quantify the true extent of this public health challenge, we need more rigorous, well-designed epidemiological studies across diverse regions. This will enable informed decision making and resource allocation for those in need.

## Introduction

Natural disasters have severe consequences for individuals and societies. They stem from atmospheric, geologic or hydrologic hazards that are partly influenced by anthropogenic climate change and have effects that are mediated by human-made social, economic and environmental conditions (WHO, 2006). The frequency and intensity of climate extremes are at an all-time high and expected to further increase (IPCC, 2023). With over three billion people living in contexts that are highly vulnerable to climate change-related events, these events represent one of the most pressing global public health challenges (IPCC, 2023; United Nations Office for the Coordination of Humanitarian Affairs, 2020). In the aftermath of natural hazards, people may experience direct (e.g., loss of significant others or physical injury) as well as indirect consequences (e.g., forced migration) that, in turn, contribute to mental health complaints.

Developing countries disproportionately bear the negative impacts of climate change and natural hazards (IPCC, 2023; WHO, 2006), and are at the same time severely disadvantaged in the availability of mental health resources (WHO, 2021). Natural hazards can thus exacerbate existing disparities (Clayton *et al.*, 2021). Additionally, these countries are more frequently affected by armed conflicts (Davies *et al.*, 2024). It is therefore likely that the prevalence of mental disorders in populations exposed to natural hazards from the Global South is higher relative to the Global North. Yet, most studies on mental health outcomes following exposure to natural hazards have been conducted in the Global North, primarily the United States. A synthesis of research on mental health outcomes following natural hazards in the Global South is critical to identify research gaps and to inform decision making and resource allocation. Previous reviews and meta-analyses have only focused on single events in the Global South (e.g., Cénat *et al.*, 2020; Hosseinnejad *et al.*, 2022) or narratively summarised available evidence on the impact of weather extremes in the Global South (e.g., Deglon *et al.*, 2023; Rataj *et al.*, 2016). However, there is no quantitative estimate of the mental health burden following natural hazards.

To this end, we conducted a meta-analysis on prevalences of mental disorders following natural hazards in the Global South. We report short- and long-term prevalences in the general population and among specific subgroups and consider the role of risk of bias, recent conflict history and type of natural hazard.

## Methods

This meta-analysis is part of a larger pre-registered project in the International Prospective Register of Systematic Reviews (CRD42023396622) and follows the Preferred Reporting Items for Systematic Reviews and Meta-Analyses guidelines (Page *et al.*, 2021). Minor deviations from the protocol are detailed in Supplementary Table S1.

### Literature search

We conducted multi-field searches (in titles, abstracts and key concepts) in MEDLINE, PsycINFO and Web of Science covering all literature from database inception to February 13, 2024. This electronic database search was supplemented with a MeSH search in the MEDLINE database on the same date. Terms indicative of natural hazards were combined with terms indicative of mental health. These terms were based on previous systematic reviews and meta-analyses in this field (Beaglehole *et al.*, 2018; Cuijpers *et al.*, 2023; Newnham *et al.*, 2022) as well as content-related considerations. A detailed table of the search terms can be found in the Supplementary Table S2. The results of the database search were loaded into Rayyan, a web tool for systematic reviews (Ouzzani *et al.*, 2016). We furthermore screened Google Scholar and reference lists from other reviews and primary studies, and compared the results with potentially eligible known studies. We applied no restrictions on publication status or language.

### Inclusion criteria and selection of studies

Studies were included if they (a) reported on the prevalence of one or more mental disorders according to the International Classification of Diseases (ICD) or Diagnostic and Statistical Manual of Mental Disorders (DSM) criteria, (b) as assessed with a validated semi-structured interview or self-report questionnaire, (c) at least 1 month after onset of the hazard; (d) included participants in the *Global South*, (e) with a mean age of 18 years or older, (f) who were affected by any kind of natural hazard or weather extreme; (g) applied a cross-sectional or longitudinal observational study design with or without a non-exposed control group and (h) included at least 100 participants. Suicidality, though not a clinical diagnosis itself, was included because of its high clinical relevance if assessed with more than one item. We did not restrict the types of natural hazards. We classified countries as *Global South* based on the Human Development Index (HDI) by the United Nations Development Programme (2023). The HDI classifies countries into very high (Global North), high, medium or low human development (Global South) based on life expectancy at birth, years of schooling and the gross national income per capita. As of 2022, 125 countries fall within the lower three categories (United Nations Development Programme, 2023). China was excluded from the current study due to recent rapid development therein, and because the substantial number of studies conducted there would have had inordinate influence on the review’s findings and conclusions (e.g., studies on the 2008 Wenchuan earthquake comprised over 50% of studies in a previous meta-analysis on prevalence of post-traumatic stress disorder (PTSD) after earthquakes; Dai *et al.*, 2016).

We excluded studies with samples (a) exposed to a technological disaster, agricultural pest or pandemics; (b) with an occupational risk of high degree exposure to adverse consequences such as firefighters, rescue workers or clinical health staff; (c) acquired through mental health clinics or psychological support groups; (d) that involved tourist groups who visited respective countries and returned home after the event; (e) studied with an experimental or qualitative study design and (f) retrospectively reporting symptom severity before the event. Cross-sectional studies with exclusively exposed samples were required to report on the number of study participants who scored above a cut-off value or fulfilled diagnostic criteria. Longitudinal studies or those including both exposed and non-exposed comparison groups were considered eligible provided they adequately reported case numbers or severity scores for each assessment or group.

After removing duplicates, four independent reviewers assessed title and abstract from a subsample of all hits. This procedure was chosen given the large number of hits and guided by the AMSTAR 2 appraisal tool for the quality of systematic reviews and meta-analyses (Shea *et al.*, 2017). The intraclass correlation coefficient between all four reviewers was 0.76 with an agreement rate of 84.7%. All disagreements were solved through consensus and the remaining studies were assessed by one of the reviewers with unclear cases being discussed with all reviewers. For the full-text assessments, the same procedure was applied. Two independent reviewers assessed the eligibility of 15% of studies. The intraclass correlation was 0.82 with an agreement rate of 91.4%. The remaining studies were consequently assessed by one reviewer, with unclear cases discussed with both reviewers. Articles published in languages other than English, Spanish or French were assessed with the help of translators.

### Coding of study characteristics

Data extraction was completed by two independent reviewers with any disagreements resolved by consensus. Extracted data included general publication details, information on the event, the sample and any outcomes. We contacted authors up to twice in case of missing data (seven studies) and obtained additional information for two studies (Bailey *et al.*, 2010; Dahal *et al*., 2018). Outcome data were classified into short-term (within 12 months) and long-term (any later assessment) point prevalence data based on the time passed between the onset of the natural hazard and the assessment. In case of multiple short- or long-term assessments, we included data from the later assessments.

Guided by the definition of the US National Oceanic and Atmospheric Administration (2024), we classified hurricanes, typhoons and cyclones in our analyses under the term *tropical cyclone* as they describe the same weather phenomenon.

**Figure 1.**
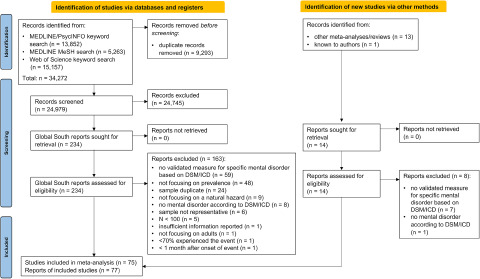
Study selection process.

### Risk of bias assessment

Due to the heterogeneity of included study designs, we developed a risk of bias appraisal tool based on the Risk of Bias Assessment Tool for Non-randomized Studies (Kim *et al.*, 2013), the Mixed Methods Appraisal Tool (Hong *et al.*, 2018) and the Strengthening the Reporting of Observational Studies in Epidemiology guidelines (von Elm *et al.*, 2007) including previously applied modifications (Morina *et al.*, 2018). The tool considers different study designs and includes selection, performance, attrition and reporting biases caused by (1) inadequate participant selection; (2) inadequate consideration of confounding variables; (3) inadequate outcome measurements; (4) inadequate handling of incomplete outcome data and (5) selective reporting of outcomes. Confounding variables included multiple exposure to natural hazards or other mass-scale traumatic events such as armed conflict. The full appraisal tool is presented in Supplementary Table S3. After piloting of the rating procedure, three independent raters assessed risk of bias of each included study and all disagreements were discussed until consensus was reached (intraclass correlation coefficient 0.82, 95% CI 0.79–0.85).

### Analysis strategy

All analyses were conducted with RStudio 2023.06.2 (Posit team, 2023) with the packages meta v.6.5-0 (Schwarzer *et al.*, 2015) and dmetar v.0.1.0 (Harrer *et al.*, 2019). We used generalised linear mixed models based on the logit transformation to avoid misleading results in the analysis of proportions (Schwarzer *et al.*, 2019). For aggregated severity scores, we used Hedges’ *g* to correct for small-sample bias (Harrer *et al.*, 2021). We conducted random-effects meta-analyses to account for expected between-study heterogeneity regarding outcome measure, natural hazard or study location (Borenstein *et al.*, 2021). Heterogeneity was assessed using the *Q* statistic and Higgins’ *I*^2^ (Higgins *et al.*, 2003). Possible publication bias was assessed for all analyses that included more than 10 studies (Sterne *et al.*, 2011) using Egger’s test (Egger *et al.*, 1997). To explore potential sources of heterogeneity, subgroup analyses were conducted with a minimum of four studies per group. Further sensitivity analyses were conducted for the main analyses with the leave-one-out approach (Viechtbauer and Cheung, 2010) and by calculating outlier-adjusted effect sizes excluding all studies whose 95% CI did not overlap with the 95% CI of the pooled effect.

## Results

After screening 24,979 titles and abstracts and 248 full texts of studies conducted in Global South countries, we included 77 reports of 75 cross-sectional studies (six included a non-exposed control group), with overall 82,400 participants (see Fig. 1for the study selection process). We found no reports of pre-post studies. A list of excluded studies with reasons for exclusion can be found in the Supplementary Table S4.

### Overview of studies on the mental burden after natural hazards

**Figure 2.**
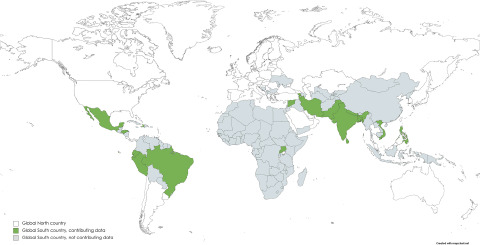
Global South countries according to the United Nations Human Development Index (HDI) as of 2022.

Studies were conducted in 17 Global South countries (13.6% of all Global South countries; see Fig. 2), comprising three countries of low (9.4%), six countries of medium (13.6%) and eight countries of high human development (16.3%). A total of 50 reports on studies from China (36 on the 2008 Wenchuan earthquake) were not considered in this meta-analysis. Publications were written in English (96.1%), Farsi (2.6%) and Spanish (1.3%). Most studies were conducted after the 2015 Gorkha earthquake in Nepal (13.0%), the 2005 Kashmir earthquake in Pakistan (10.4%) and the 2010 earthquake in Haiti (9.1%). A total of 44 studies were conducted after earthquakes (57.1%), followed by floods and related mudslides (14.3%) and tropical cyclones (13.0%). The death toll thereby ranged from 0 (the 2009 flood in Brazil) to over 200,000 (the 2004 Indian Ocean tsunami; see Supplementary Table S5). The mean age of the samples was 38.1 years and 38.1% received only primary education or less. 65.1% identified as female, 34.9% as male and only one person overall indicated ‘third gender’. Study details are presented in Table 1.

**Table 1.** Characteristicsof included studies

Study	Event, Location	Time since event (in months)	Total sample size	Population	Mean age (SD)	Outcomes	Instrument
**Cross-sectional studies with exposed samples only**							
Abdollahi *et al.* (2011)	2005 Zarand earthquake, Iran	24	172	University sample	24.2 (7.8)	PTSD	PDS
Acharya Pandey *et al.* (2023)	2015 Gorkha earthquake, Nepal	36	1076	General population	40.5 (13.4)	PTSD	PCL-C
Adhikari Baral and Bhagawati (2019)	2015 Gorkha earthquake, Nepal	10	291	General population	43.6 (14.4)	PTSD	PCL-5
Ali *et al.* (2012)	2005 Kashmir earthquake, Pakistan*	31	300	Displaced individuals	37.8 (14.0)	PTSD	DTS
Amstadter *et al.* (2009)	2006 typhoon Xangsane/Milenyo, Vietnam	2.5	797	General population	41.6 (16.5)	PTSD, MDD, Panic Disorder, GAD	NWS-PTSD, SCID
Anwar *et al.* (2013)	2005 Kashmir earthquake, Pakistan*	47.5	387	Married women of reproductive age	33.2 (7.2)	PTSD	HTQ
Bailey *et al.* (2010)	1988 Spitak earthquake, Armenia	162	200	Members of 12 families	n.r.	PTSD, Alcohol use disorder	PTSD-RI, MINI
Bandla *et al.* (2019)	2015 Chennai floods, India	2	223	General population	n.r.	PTSD, MDD	PCL-C, BDI
Blanc *et al.* (2016)	2010 earthquake, Haiti	27	167	General population	30.6 (11.0)	PTSD, MDD	PCL-S, BDI-II
Burnett and Helm (2013)	2010 earthquake, Haiti	4.5	111	University sample	23.7 (5.1)	PTSD	PCL-C
Cairo *et al.* (2010)	2007 Pisco earthquake, Peru	5	298	General population	41.2 (15.4)	PTSD	PCL-C
Cénat and Derivois (2014)	2010 earthquake, Haiti	30	1355	General population	31.6 (14.4)	MDD	BDI-II
Cerdá *et al.* (2013)	2010 earthquake, Haiti	2.75	1313	General population & displaced individuals	n.r.	PTSD, MDD	PCL, PHQ-9
Charak *et al.* (2014)	2010 Ladakh floods, India	8	200	General population	34.8 (13.7)	PTSD	PCL-S
Cherian *et al.* (2020)	2018 Kerala floods, India	13	100	General population	n.r.	PTSD	PCL-5
Contreras *et al.* (2018)	2017 El Niño Costero floods, Peru	n.r.	116	Informal settlements	n.r.	MDD	PHQ-9
Dahal *et al*. (2017)	2015 Gorkha earthquake, Nepal	9	535	General population	36.3 (13.2)	PTSD	PCL-C
Demirchyan *et al.* (2022)	1988 Spitak earthquake, Armenia	276	722	Employees of the Ministry of Health + relatives	58.4 (12.1)	PTSD, MDD	PCL-C, DIS-III
Dévieux *et al.* (2013)	2010 earthquake, Haiti	n.r.	104	HIV-positive individuals	34.2 (9.7)	PTSD	PCL-C
Divsalar and Dehesh (2020)	2003 Bam earthquake, Iran	144	1500	General population	40.2 (13.6)	PTSD, MDD	PCL-C, BDI -II
Espinoza-Neyra *et al.* (2017)	2017 El Niño Costero floods, Peru	1.5	184	General population	n.r.	PTSD	DTS
Farhoudian *et al.* (2006)	2003 Bam earthquake, Iran	8	786	General population	31.9 (13.4)	PTSD	CIDI
Feder *et al.* (2013)	2005 Kashmir earthquake, Pakistan*	36	200	General population	37.7 (11.7)	PTSD	TSSC
Flores *et al.* (2014)	2007 Pisco earthquake, Peru	48	1012	General population	43.1 (n.r.)	PTSD	PCL-C
Gay *et al.* (2020)	Several Popocatépetl volcanic eruptions, 1999 Teziutlán floods and mudslide, Mexico Several Tungurahua volcanic eruptions, Ecuador	108 24	727	General population	38.5 (14.6)	PTSD	CIDI
Ghaffari-Nejad *et al.* (2007)	2003 Bam earthquake, Iran	n.r.	400	General population	37.8 (12.7)	PGD	ICG
Goenjian *et al.* (2021)	1988 Spitak earthquake, Armenia	300	109	Follow-up of school sample	38.0 (n.r.)	PTSD	PCL-5
Goodarzi *et al.* (2011)	2003 Bam earthquake, Iran	n.r.	117	Selected employees	30.5 (8.4)	PTSD	Mississippi Post-Traumatic Stress Scale
Hagh-Shenas *et al.* (2006)	2003 Bam earthquake, Iran	1.5	145	General population	31.8 (n.r.)	PTSD	PSS-SR
Hashmi *et al.* (2011)	2005 Kashmir earthquake, Pakistan*	7	361	Displaced individuals	35.0 (13.8)	PTSD	PCL
Horiguchi and Nakazawa (2021)	2013 typhoon Yolanda/Haiyan, Philippines	38	296	Mothers of young children	n.r.	PTSD	PCL-5
Howard *et al.* (1999)	1991 Mount Pinatubo volcanic eruption, Philippines	72	351	Displaced individuals	n.r.	PTSD, MDD, Panic Disorder, GAD	PCL-S, PRIME-MD
Jha *et al.* (2017) Sample 1 (low exposure) Sample 2 (high exposure)	2015 Gorkha earthquake, Nepal	3 11	159 203	General population	n.r.	PTSD	PCL-5
Kabunga *et al.* (2022)	Several landslides on Mount Elgon, Uganda	Mixed	587	General population	n.r.	PTSD	PCL-C-5
Kane *et al.* (2018)	2015 Gorkha earthquake, Nepal	4	513	General population	42.1 (15.8)	PTSD, Suicidality	PCL-C, CIDI
Kar *et al.* (2014)	2004 Indian Ocean tsunami, India	55	666	General population	42.8 (n.r.)	PTSD, Suicidality	SRS-PTSD, Suicidality questionnaire
Kohn *et al.* (2005), Kohn (2013)	1998 hurricane Mitch, Honduras	2.5, 24	800 (follow-up 604)	General population	37.0 (16.8)	PTSD	CIDI
Kumar *et al.* (2007)	2004 Indian Ocean tsunami, India	2	314	General population	35.8 (14.0)	PTSD	HTQ
Labarda *et al.* (2020)	2013 typhoon Yolanda/Haiyan, Philippines	48	345	Socioeconomically disadvantaged households	42.0 (13.1)	PTSD	PCL-5
Lommen *et al.* (2009)	2004 Indian Ocean tsunami, Sri Lanka	15	113	Displaced individuals	35.9 (10.1)	PTSD	PDS
Mamun *et al.* (2019)	2017 cyclone Mora, Bangladesh	4	111	Women	40.1 (13.6)	MDD	PHQ-9
Mamun *et al.* (2021)	2019 floods, Bangladesh	4.5	348	General population	45.5 (14.9)	MDD, GAD, Suicidality	PHQ-9, GAD-7, Suicidality questionnaire
Maya-Mondragón *et al.* (2019)	2017 Puebla earthquake, Mexico	1.5	44855	Waitlist from general health clinics	n.r. (17.0)	PTSD, MDD	SQD
Miyazaki *et al.* (2006)	2004 Indian Ocean tsunami, Sri Lanka	n.r.	127	General population	39.0 (13.9)	PTSD	HTQ
Mordeno and Hall (2017)	2011 typhoon Washi/Sendong, Philippines	n.r.	500	Displaced individuals	28.5 (12.6)	PTSD	PCL-5
Mordeno *et al.* (2017)	2013 typhoon Yolanda/Haiyan, Philippines	n.r.	664	University sample	18.0 (1.8)	PTSD	PCL-5
Muldoon *et al.* (2017)	2015 Gorkha earthquake, Nepal	6	375	General population	35.0 (n.r.)	PTSD	PCL-C
Naeem *et al.* (2011)	2005 Kashmir earthquake, Pakistan*	18	1291	Displaced individuals	32.9 (n.r.)	PTSD	TSSC
Niaz *et al.* (2006)	2005 Kashmir earthquake, Pakistan*	4	155	University sample & displaced individuals	n.r.	PTSD	TSSC
Norris *et al.* (2001)	1997 hurricane Paulina, Mexico	6	200	General population	45.3 (n.r.)	PTSD	Revised Civilian Mississippi Scale
Norris *et al.* (2004)	1999 floods, Mexico	12, 24	561	General population	37.3 (n.r.)	PTSD, MDD	CIDI
Pollack *et al.* (2016)	Several typhoons, tropical storms, and floods, Vietnam	n.r.	1000	General population	n.r.	PTSD, MDD, GAD	PDS, PHQ-9, GAD-7
Rajkumar *et al.* (2015)	2004 Indian Ocean tsunami, India	9	643	General population	33.7 (14.5)	PGD	Complicated Grief Assessment
Rana *et al.* (2008)	2005 Kashmir earthquake, Pakistan*	8	111	Displaced individuals	n.r.	PTSD	TSSC
Ranasinghe and Levy (2007)	2004 Indian Ocean tsunami, Sri Lanka	6	264	Displaced individuals	38.4 (14.5)	PTSD	PTSD Symptom Scale-Interview
Ranasinghe *et al.* (2023)	2004 Indian Ocean tsunami, Sri Lanka	96	325	General population	46.9 (15.8)	PTSD	PTSD Symptom Scale-Interview
Reis *et al.* (2016)	2009 December floods, Brazil	51	113	General population	37.5 (12.1)	PTSD	DTS
Schwind *et al.* (2019)	2015 Gorkha earthquake, Nepal	12	223	General population	45.1 (16.6)	MDD, PTSD	BDI-II, PCL-C
Sharma *et al.* (2023)	2015 Gorkha earthquake, Nepal	84	124	General population	n.r.	PTSD	PCL-5
Silvestre *et al.* (2014)	2010 earthquake, Haiti	22.5	246	University sample	23.1 (3.1)	PTSD	PSS-I
Soqia *et al.* (2023)	2023 Turkey-Syria earthquake, Syria	1.5	1406	General population	28.6 (8.9)	PTSD, MDD, GAD	PCL-5, PHQ-9, GAD-7
Srivastava *et al.* (2015)	2013 Uttarakhand floods, India	12	1651	General population	40.0 (n.r.)	PTSD	PCL-C
Thapa *et al.* (2018)	2015 Gorkha earthquake, Nepal	14	198	General population	35.1 (18.0)	PTSD	PTSD-8
Tiyuri *et al.* (2023)	2019 Spring floodss, Iran	5.5	1671	General population	n.r.	MDD	PHQ-9
Valladares-Garrido *et al*. (2022a, 2022b)	2021 Sullana earthquake, Peru	1.5	177	General population	n.r.	PTSD, GAD	PCL-C, GAD-7
Wagenaar *et al.* (2012)	2010 earthquake, Haiti	16	408	General population	n.r.	MDD	BDI-II
Wagle *et al.* (2021)	2015 Gorkha earthquake, Nepal	23	362	Elderly	70.6 (8.1)	PTSD, MDD	SQD
Wani *et al.* (2020)	2014 Kashmir floods, India*	3	500	General population	40.7 (10.0)	PTSD	PCL-C
Zuñiga *et al.* (2019)	2017 Chiapas and Puebla earthquake, Mexico	2.5	1539	General population	36.8 (12.7)	PTSD	DTS
**Cross-sectional studies with exposed vs. non-exposed sample**							
Study	Event	Time point assessed (in months)	Total sample size	Population	Mean age (SD)	Outcomes	Instrument
Amiri *et al.* (2022) Exposed Non-exposed	2003 Bam earthquake, Iran	204	574 244	General population	46.6 (11.5)	Substance use: tobacco, sedatives, other drugs	ASSIST
Goenjian *et al.* (1994) Exposed Non-exposed	1988 Spitak earthquake, Armenia	18	66 59	General population	26.0 (n.r.) 41.0 (n.r.)	PTSD	PTSD-RI
Najarian *et al.* (2017) Exposed Non-exposed	1988 Spitak earthquake, Armenia	240	107 37	General population	38.0 (n.r.) 39.0 (n.r.)	PTSD	PTSD-RI
Perera-Diltz (2006) Exposed Non-exposed	2004 Indian Ocean tsunami, Sri Lanka	17	384 54	General population	40.8 (14.5) 39.5 (14.6)	PTSD	IES-R
Ramachandran *et al.* (2006) Exposed Non-exposed	2004 Indian Ocean tsunami, India	4.75	1115 1103	General population	41.7 (13.2) 39.2 (12.9)	PTSD	HTQ
Toukmanian *et al.* (2000) Exposed Non-exposed	1988 Spitak earthquake, Armenia	12	158 302	General population	41.1 (n.r.)	MDD	BDI-II

Note: ASSIST = Alcohol, Smoking and Substance Involvement Screening; BDI = Beck Depression Inventory; CIDI = Composite International Diagnostic Interview; DIS = Diagnostic Interview Schedule; DTS = Davidson Trauma Scale; GAD = generalised anxiety disorder; GAD-7 = Generalised Anxiety Disorder Scale-7; HTQ = Harvard Trauma Questionnaire; ICG = Inventory of Complicated Grief; IES = Impact of Events Scale; IES-R = Impact of Events Scale-Revised; MINI = Mini International Neuropsychiatric Interview; MDD = major depressive disorder; PCL = PTSD Checklist; PCL-5 = PTSD Checklist for DMS-5; PCL-C = PTSD Checklist-Civilian Version; PCL-S = PTSD checklist-specific; PDS = Post-traumatic Diagnostic Scale; PGD = prolonged grief disorder; PHQ-9 = Patient Health Questionnaire-9; PSS-I = PTSD Symptom Scale-Interview; PSS-SR = PTSD Scale-Self Report; PTSD-8 = Post-traumatic Stress Disorder-8; PTSD-RI = PTSD Reaction Index; SCID = Structured Clinical Interview for DSM Disorders; SQD = Screening Questionnaire for Disaster Mental Health; SRS-PTSD = Self-Rating Scale for PTSD; PTSD-RI = University of California, Los Angeles PTSD Reaction Index.

* The countries were named according to what was reported in the respective publication.

### Prevalence of mental disorders in the general population

**Figure 3.**
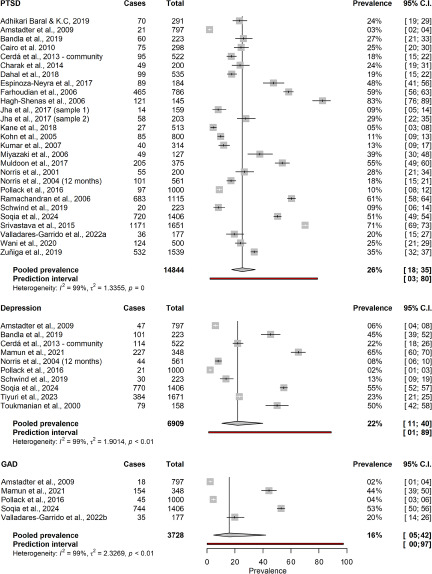
Short-term prevalence of mental disorders. C.I. = confidence interval. GAD = generalised anxiety disorder. PTSD = posttraumatic stress disorder.

**Figure 4.**
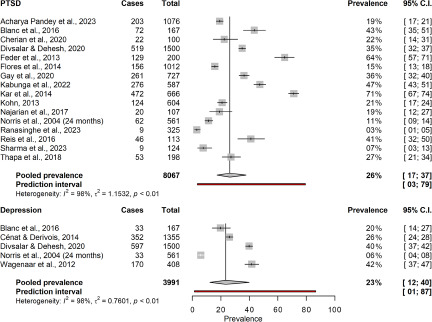
Long-term prevalence of mental disorders. C.I. = confidence interval. PTSD = posttraumatic stress disorder.

During the first year following the onset of a natural hazard (mean time 5.6 months), the aggregated point prevalence of PTSD in the general population was estimated at 26.0% (95% CI 18.5–36.3; *k* = 27; *n* = 14,844; see Fig. 3 for all short-term prevalence estimates). An equivalent prevalence of 26.0% (95% CI 17.1–37.4) was found for long-term assessments on average 5 years after onset of the hazard (range 1–20 years). This analysis included 16 studies and 8,067 individuals (see Fig. 4 for all long-term prevalence estimates). The aggregated point prevalence for major depressive disorder in the general population was 21.7% during the first year after onset of the hazard (95% CI 10.5–39.6; *k* = 10; *n* = 6,909). Assessments took place on average 6.3 months after the onset. The prevalence remained stable at 23.4% (95% CI 12.3–39.8; *k* = 5; *n* = 3,991) for an average of 4 years after the onset (range 1.5–12 years). Generalised anxiety disorder (GAD) was only assessed as short-term outcome (mean 3.6 months). Analyses across 5 studies and 3,728 individuals yielded an overall prevalence of 15.9% (95% CI 4.7–42.0). The heterogeneity between studies in the main analyses was considerable with *I*^2^ ranging from 93.3% to 99.4% (all *p*’s < .001).

The presence of other mental disorders was assessed in a limited number of studies. These studies found a prevalence of 9.3% for panic disorder 3 months after a typhoon (Amstadter *et al.*, 2009) and a prevalence of 9.0% for insomnia 2 months after an earthquake (Valladares-Garrido *et al.*, 2022b). Two studies assessed the prevalence of prolonged grief disorder and found prevalence estimates of 14.2% (Rajkumar *et al.*, 2015) and 76.0% (Ghaffari-Nejad *et al.*, 2007) within a year after a tsunami and an earthquake, respectively. The range in the findings on suicidality was substantial. Suicidal thoughts after exposure to a natural hazard were present in 10.9% (Kane *et al.*, 2018), 24.6% (Kar *et al.*, 2014) and 57.5% (Mamun *et al.*, 2021) of survivors. Similarly, suicide plans were present in 1.0% (Kane *et al.*, 2018), 5.7% (Mamun *et al.*, 2021) and 17.3% (Kar *et al.*, 2014) of survivors. Finally, suicide attempts were reported by 0.2% (Kane *et al.*, 2018), 2.0% (Mamun *et al.*, 2021) and 6.5% (Kar *et al.*, 2014) of survivors.

### Prevalence of mental disorders in (temporarily) displaced survivors

The prevalence of PTSD in temporary shelter camps (*k* = 6; *n* = 2,661) was 46.5% (95% CI 39.0–54.2). At the time of the assessment, survivors resided in the camps for 3–18 months. In two studies, it was reported that the temporary villages consisted of tents. Three studies included permanently displaced individuals. Howard *et al.* (1999) found a prevalence of 27.6% for PTSD, 14.0% for depression, 6.0% for panic disorder, 3.1% for GAD, 0.3% for bipolar disorder and 7.4% for dysthymia in relocated individuals 6 years after a volcanic eruption. The remaining studies reported a PTSD prevalence of 41.3% 2.5 years after an earthquake (Ali *et al.*, 2012) and of 54.6% 4.5 years after a typhoon (Mordeno and Hall, 2017). Findings from studies focusing on other specific subgroups are summarised in Supplementary Figure S1)

### Previous exposure to armed conflicts

The majority of armed conflicts in the past decades were located in countries of the Global South (Davies *et al.*, 2024), making it inevitable to consider previous human-made traumatic events that samples may have experienced. Almost forty percent (29/75) of the included studies were conducted in post- or active-conflict settings (i.e., at least 1,000 people died in the region from armed conflicts within a year of the sample’s mean lifetime, Davies *et al.*, 2024). Yet, the impact of previous exposure to armed conflicts could only be assessed for PTSD, which yielded no significant influence (see Supplementary Table S6).

### Moderating effects according to type of natural hazard

The prevalence of PTSD within a year after onset of the natural hazard varied significantly depending on the type of natural hazard (*p* = .019). The lowest prevalence was observed in the aftermath of tropical cyclones (9.7%; 95% CI 4.1–21.0), followed by earthquakes (27.7%; 95% CI 17.2–41.3) and floods (34.3%; 95% CI 21.1–50.3). This differentiation was not possible for other outcomes and long-term assessments due to the low number of studies.

### Findings from studies including a non-exposed control group

Only six studies included a non-exposed control group, and the heterogeneity of reported outcomes prohibited meta-analysis. The included studies found significantly higher symptoms of PTSD (*g* = 1.89 [Perera-Diltz, 2006] and *g* = 0.70 [Goenjian *et al.*, 1994]) as well as depression (*g* = 0.61 [Toukmanian *et al.*, 2000]) in the exposed group. Yet, the comparison of prevalence remains inconclusive. Whereas the odds for depression appeared significantly increased in the exposed group (*OR* = 3.24 [Toukmanian *et al.*, 2000]), no significant effect was detected for substance use (Amiri *et al.*, 2022) and the results for PTSD were mixed with one significant (Ramachandran *et al.*, 2006) and one non-significant study (Najarian *et al.*, 2017). However, it should be noted that the non-significant study was conducted 20 years after the earthquake and disproportionately included unexposed individuals.

### Risk of bias of included studies

The risk of bias across studies was noteworthy. The vast majority (92%) of included studies followed a cross-sectional design without a control group. Among these studies, only 7% used a rigorous probability sampling method. Instead, almost 80% of studies used self-referral methods, recruited only at limited institutions, focused on specific subgroups (e.g., university students), focused only on most affected districts, applied critical exclusion criteria (e.g., no personal loss from the event), or achieved a response rate of ≤70%. Almost half of the studies (48%) included individuals from regions affected by armed conflicts or additional natural hazards in recent years without taking into account previous traumatic experiences in their analyses. The validation status for the translation of instruments was insufficient or unclear in 80% of cases. Furthermore, information on the extent or handling of missing outcome data was limited, and no study reported on a publicly available study protocol. The risk of bias in the six studies with a non-exposed control group was slightly reduced due to adequate selections of control groups and corresponding matching of confounding variables. Detailed results of the risk of bias assessment can be found in Supplementary Table S7.

### Sensitivity analyses

Only analyses on PTSD outcomes included a sufficient number of studies to detect possible publication bias. Egger’s test was significant in both cases, suggesting possible publication bias. Leave-one-out analyses displayed several influential studies and yielded adjusted point prevalence estimates for PTSD of 24–28% within a year after the onset of the hazard. Omitting all studies whose 95% confidence interval did not overlap with the confidence interval of the aggregated prevalence resulted in a reduced prevalence of 24.8% (95% CI 21.7–28.1). Regarding the long-term point prevalence of PTSD, the estimate ranged from 26% to 29%. The outlier-adjusted estimate was 26.6% (95% CI 21.1–32.9). Given the large heterogeneity between studies on depression outcomes, the influence of individual studies was even more extensive. Omitting individual studies from analyses resulted in a short-term prevalence of 18–27% and a long-term prevalence of 20–31%. Outlier-adjusted prevalence estimates were 24.2% (95% CI 15.2–36.4) and 31.4% (95% CI 23.1–41.1) for short- and long-term assessments, respectively. Finally, leave-one-out analyses on GAD resulted in estimates between 11% and 24% and the outlier-adjusted prevalence was 17.3%. Plots of the sensitivity analyses are presented in Supplementary Figures S2–S6.

## Discussion

We meta-analysed data across 75 studies with over 80,000 individuals to estimate the prevalence of mental disorders in Global South populations affected by natural hazards. We found high point prevalences for PTSD, depression and GAD in the general population, with the former two remaining alarmingly high 4–5 years post-hazard. Prevalence estimates were even higher among displaced survivors. Although numerous countries from the Global South have been affected by armed conflicts in the past years, residence in a post-conflict setting did not moderate our findings. Compared to previous meta-analyses that mainly included studies conducted in Western countries, we note considerable differences in findings on PTSD after floods (Chen and Liu, 2015) (higher in the Global South) and tropical cyclones (Wang *et al.*, 2019) (lower in the Global South), but not after earthquakes (Dai *et al.*, 2016). Meta-analytic findings on other mental health disorders are limited.

The prevalence of disaster-related PTSD (both human-made and ‘natural’) reported in the World Mental Health (WMH) Surveys is a fraction of what was collectively found in the reviewed studies from the Global South. Across eight studies in the WMH Surveys (six of which were conducted in high-income countries), the aggregated prevalence was 2.5% (Bromet *et al.*, 2017), while we identified a prevalence of 26.0%. Future research needs to investigate potential factors that might explain this discrepancy, such as the level or type of exposure to natural hazards and pre-existing discrepancies in prevalence across different world regions. Previous literature suggests that survivors of human-made traumatic events are more likely to develop PTSD compared to survivors of natural hazards (Bromet *et al.*, 2017). The non-significant influence of previous conflicts thus appears counter-intuitive. It is nonetheless essential to bear in mind that natural hazards increase the likelihood of exposure to cascading effects such as increased intimate partner violence (Bell and Folkerth, 2016) and that all of our findings stem from cross-sectional studies that do not allow for any causal attribution for the high prevalence. Our findings suggest that there is an immense long-term mental burden in communities from the Global South affected by natural hazards, which highlights the need for mental health support in the aftermath. Shortage of healthcare professionals, limited financial resources, lack of mental health policies or stigmatisation of mental health complaints all pose challenges on the provision of mental health services in Global South countries (WHO, 2021). There is yet an urgent need to overcome the global treatment gap. This appears especially important considering that (1) many survivors below the clinical cut-offs may still experience subclinical symptoms (Brand *et al.*, 2018), (2) individuals suffering from PTSD most likely fulfil the criteria for at least one additional diagnosis, most frequently depression, anxiety disorder or substance use disorder (Rytwinski *et al.*, 2013) and (3) mental health complaints are associated with severe functional impairment (Norris *et al.*, 2002). As natural hazards and climate extremes may increase migratory movements (Hoffmann *et al.*, 2020) and are likely to become more frequent and severe (IPCC, 2023), this is a health issue of global concern.

### Limitations and future research

Future research must take the following observations into consideration. First, only 17 of 125 Global South countries are represented in our analyses with only 3 out of 32 countries of low human development, and only one study from the African continent. We furthermore excluded China due to its overall rapid recent development, although it is possible that some studies were conducted in less developed areas of the country. This limits the generalisability of our findings to all developing countries. Second, studies in the aftermath of earthquakes appear over-represented compared to their actual frequency (Centre for Research on the Epidemiology of Disasters, 2023), whereas the impact of heatwaves and droughts on mental health in the Global South appears understudied despite their frequent occurrence (IPCC, 2023). A recent meta-analysis on the association between high ambient temperatures and mental health supports the need to investigate the impact of temperature-related natural hazards on mental health in the most vulnerable regions of the world, including the African continent (Liu *et al.*, 2021). Third, it is imperative to improve the quality of research and reduce risk of bias, which was alarmingly high across the included studies. Disaster- or crisis-related studies face several challenges that present barriers to conducting high-quality research. Given the unpredictability of most natural hazards, studies are often developed and implemented at short notice, which may explain why we could not find any publicly available study protocols. Probability sampling may be complicated by inaccessibility of severely affected or remote regions, widespread impacts of events and population displacement. On the other hand, several of the included studies selected participants randomly, but restricted the inclusion to the most severely affected individuals, which is likely to bias findings of the overall effects upwards. The overall large heterogeneity between studies may only be partly attributed to methodological differences. The included moderators insufficiently explained between-study heterogeneity, which limits the validity of overall results. Fourth, less than 20% of studies used a validated translation of the applied diagnostic tool. There has been extensive controversy in the past years on the global validity of Western diagnoses and assessments or the universality of mental health (Michalopoulos *et al.*, 2015). To follow an inclusive perspective on mental health, future research should aim to develop and validate multi-language and culture-sensitive diagnostic tools that allow for comparison of outcomes while considering culture-bound idioms of distress (Kaiser and Jo Weaver, 2019). Fifth, it should be the long-term aim to shift data collection from cross-sectional observational surveys after acute events to panel and longitudinal data collections in regions frequently affected by natural hazards. This would allow for the inclusion of pre-event mental health data and subacute or chronic events such as droughts, thus contributing to a better understanding of the link between natural hazards and mental health. Finally, it is important to include control groups that have not been exposed to the natural hazard to control for additional experiences such as armed conflicts.

## Conclusions

The findings from this meta-analysis are a call for action. The high prevalence estimates of mental disorders and the associated severe functional impairment demand a resolution of the global treatment gap. It requires the implementation of mental health policies and the training of healthcare professionals, the reduction of mental health stigma and the development of low-threshold interventions in the Global South in the aftermath of natural hazards. Researchers are challenged to further investigate the impact of climatic events in low-developed countries, especially in Africa. More high-quality studies including non-exposed comparison groups are needed to obtain more valid estimates of the mental health burden.

## Supporting information

Kip et al. supplementary material Kip et al. supplementary material

## Data Availability

The dataset is available at https://osf.io/6tmjs/.

## Supplementary material

The supplementary material for this article can be found at https://doi.org/10.1017/S2045796024000672.
