# Supplementary material for: Prevalence of mental disorders in adult populations from the Global South following exposure to natural hazards: a meta-analysis
Source: Epidemiol Psychiatr Sci. 2024 Nov 28;33:e68. doi: 10.1017/S2045796024000672 (PMC11735120; doi:10.1017/S2045796024000672)

Supplementary Material

Tables

Table 1 | *Minor deviations from the protocol*

Table 2 | *Search strategies*

Table 3 | *Risk of bias appraisal tool*

Table 4 | *List of excluded studies with reasons for exclusion*

Table 5 | *Severity of studied events according to the fatality-based Universal Disaster Severity Classification*

Table 6 | *Subgroup analyses on the influence of exposure to armed conflicts*

Table 7 | *Risk of bias assessment of included studies*

Figures

Fig. 1 | *Prevalence estimates in specific subgroup*

Fig. 2 | *Plot for Leave-one-out analysis on PTSD short-term prevalence estimates*

Fig. 3 | *Plot for Leave-one-out analysis on PTSD long-term prevalence estimates*

Fig. 4 | *Plot for Leave-one-out analysis on depression short-term prevalence estimates*

Fig. 5 | *Plot for Leave-one-out analysis on depression long-term prevalence estimates*

Fig. 6 | *Plot for Leave-one-out analysis on GAD short-term prevalence estimates*

Supplementary Table 1

Minor deviations from the protocol

1	Given the epidemiological character of our meta-analysis on mental disorders, we renounced searching the Global Health as well as Clinicaltrials.gov databases
2	Because of the vast number of hits in the systematic search with over 30.000 articles, four instead of two reviewers were involved in the screening of title and abstract.

Supplementary Table 2

Search strategy

	Concept 1	Concept 2
**Key Terms** (title, abstract, key words)	"natural disaster*" OR "natural emergenc*" OR "natural crisis"<br>OR<br>storm OR flood* OR hurricane OR cyclone OR monsoon OR typhoon OR wildfire OR fire OR bushfire OR desertification OR drought* OR landslide OR tsunami OR earthquake OR flood OR tornado* OR hurricane OR cyclon* OR sinkhole OR "ice storm" OR heatwave OR "heat wave" OR "extreme temperature" OR avalanche OR blizzard OR "snow storm" OR "cold wave" OR thunderstorm OR "rising sea-level" OR "volcan*" OR hail	"mental disorder" OR "mental health" OR "mental disease" OR "mental illness" OR "affective disorder" OR "mood disorder" OR depress* OR bipolar OR mania OR ptsd OR "posttraumatic stress disorder" OR "post-traumatic stress disorder" OR anxiety OR phobia OR panic<br>OR psychosis OR schizophre*<br>OR "substance use disorder" OR "alcohol dependen*" OR "drug dependen*" OR alcoholism OR addiction<br>OR "eating disorder" OR "sleep disorder" OR "suicid*" OR somatoform OR "obsessive compulsive disorder" OR OCD OR "personality disorder"<br>OR "Disruptive, Impulse Control, and Conduct Disorders" OR "Feeding and eating disorder" OR "attachment disorder"
OR		
**MeSH**	Disasters/ OR<br>**exp** Natural Disasters/<br>➔ included subheadings: Avalanches, Cyclonic Storms, Droughts, Earthquakes, Floods, Landslides, Tidal Waves, Tornadoes, Wildfires	Mental Health/ OR<br>Psychophysiology/ OR<br>Stress, Psychological/ OR<br>**exp** Mental Disorders/ OR<br>Adaptation, Psychological/ OR<br>Emotional Adjustment/ OR<br>Depression/ OR<br>Psychological Distress/ OR<br>Anxiety/ OR<br>Obsessive Behavior/ OR<br>Affective Symptoms/ OR<br>Counseling/ OR<br>Directive Counseling/ OR<br>Distance Counseling/ OR<br>**exp** Emergency Services, Psychiatric/ OR<br>Social Work, Psychiatric/ OR<br>**exp** Psychotherapy

Supplementary Table 3

Risk of bias appraisal tool

**Selection of participants**<br>Selection bias caused by the inadequate selection of participants	
Low Risk	**Cross-sectional study with exposed vs. unexposed sample**<br>• the exposed and unexposed groups were selected from comparable population groups<br>• the exposed group was clearly defined and it was clearly demonstrated that the control group was not affected<br>• study did not apply critical exclusion criteria (e.g., excluded only those who were absent at the time of disaster or with mental retardation)<br>• response rate ≥ 85% (Morina 2018)<br><br>**Before-after study**<br>The study participants were consecutively recruited and the data were collected prospectively<br><br>**Cross-sectional study with exposed sample only**<br>• response rate ≥ 85% (Morina 2018)<br>→follow-up studies: rating is based on both t0 response rate from all eligible individuals and follow-up response rate<br>• study did not apply critical exclusion criteria or only very few/no participants were excluded based on critical exclusion criteria<br>• study used probability sampling of participants (including random place of sampling)<br>• sample is representative of the target population
Moderate Risk	• Criteria for low risk fulfilled, but response rate not reported<br>• Minor violations of probability sampling (e.g., excluding inaccessible districts)
High Risk	**Cross-sectional study with exposed vs. unexposed sample**<br>• the exposed and unexposed groups are not comparable population groups<br>• the participant definitions were generated by self-reported data (e.g., subjective affectedness)<br>• control group included affected individuals<br>• response rate ≤70%<br><br>**Before-after study**<br>• retrospective data collection was performed<br><br>**Cross-sectional study with exposed sample only**<br>• response rate ≤70%<br>• follow-up studies for which t0 response rate is not reported<br>• critical exclusion criteria (e.g., illiterate, medication, psychotherapy)<br>• non-probability sampling was used (convenience sample, self-selection sample, snowball sampling) including non-random selection of recruitment places<br>• sample is not representative of the target population (e.g., students)
Unclear	It is uncertain whether the selection of participants resulted in a „high risk' or a „low risk' of bias<br>• **Cross-sectional study with exposed vs. unexposed sample:** composition of control group unclear; response rate not reported<br>• **Cross-sectional study with exposed sample only:** no information on sample reported; not reported how many participants were excluded based on critical exclusion criteria

**Confounding variables**<br>Selection biases caused by the inadequate confirmation and consideration of confounding variables (mainly: additional mass-scale trauma/disaster)	
Low Risk	**Cross-sectional study with exposed vs. unexposed sample**<br>• the major confounding variables were adequately confirmed and considered during the design phase (e.g., through matching, participation restriction, or other methods)<br>• the major confounding variables were adequately confirmed and adjusted for during the analysis phase (e.g., through stratification, propensity score approaches, statistical adjustments, or other methods, e.g., random effects for geographical clusters)<br><br>**Before-after study**<br>• study reports disaster-related prevalence/ new-onset prevalence separately<br><br>**Cross-sectional study with exposed sample only**<br>• no additional mass-scale disaster/no conflict since mean birth year of study participants in region
High Risk	**Cross-sectional study with exposed vs. unexposed sample**<br>• the major confounding variables were not considered<br>• although the existence of major confounding variables was confirmed, these variables were not adequately considered during the design and analysis phases.<br><br>**Before-after study**<br>• study reports variables that might have influenced prevalence rates (e.g., psychological first aid after disaster)<br><br>**Cross-sectional study with exposed sample only**<br>• disaster in post-conflict setting (as indexed by Uppsala Conflict Database or information in primary study)<br>• additional mass-scale disaster (e.g., nuclear disaster after tsunami or second natural disaster)
Unclear	It is uncertain whether the confounding variables resulted in a „high risk' or a „low risk' of bias

**Measurement of outcome**<br>Performance biases caused by inadequate measurements of outcomes<br>(If study reports multiple relevant outcomes, multiple ratings can be given in this category)	
Low Risk	• data were obtained from trustworthy sources (e.g., suicide rates)<br>• data were obtained from (semi-)structured interviews and interviewers received an adequate training/supervision or computer-assisted interviews were applied<br>• the validity and reliability of the language-version used has been confirmed
High Risk	• data were obtained through self-reported methods (or self-report instruments applied as interviews)<br>• a clear case of interviewer bias* or no training of interviewers<br>• The measurement tool was translated and the validity of the translation has not been confirmed (pre-testing is insufficient)
Unclear	It is uncertain whether the exposure measurement resulted in a „high risk' or a „low risk' of bias, e.g., interviewers had no psychological background, the background is unclear or details of interviewer training is not reported

* "Interviewer bias" describes a situation in which the characteristics of the interviewers may affect the study results. This phenomenon can be reduced through the training of investigators.

**Incomplete outcome data**<br>Attrition biases caused by the inadequate handling of incomplete outcome data	
Low Risk	**Cross-sectional study with exposed vs. unexposed sample**<br>• There are no or few missing data (<5%) related to prevalence outcome, the missing data is similar across groups<br><br>**Before-after study**<br>• Information about the number of participants before and after the study exists and the baseline did not differ with respect to completed and failed study participants<br><br>**Cross-sectional study with exposed sample only**<br>• There are no or few missing data (<5%) related to prevalence outcome (Higgins et al., 2016, RoB2)<br><br>Overall: missingness of data is not reported in article, but outcome measure is an interview or a self-report measure applied as interview
High Risk	**Cross-sectional study with exposed vs. unexposed sample**<br>• both groups have ≥5% missing data (regardless of causes for missingness) related to prevalence outcome<br><br>**Before-after study**<br>• Differences exist with respect to the baseline for completed and failed participants<br><br>**Cross-sectional study with exposed sample only**<br>• ≤80% participants contributed to analyses (regardless of statistical compensation for non-response, e.g., imputation) (Higgins et al., 2016, RoB2)<br>• causes for missing data appear related to the true values of outcomes (e.g., participants reported questionnaire to be too distressing; missing not at random)
Unclear	It is uncertain whether the incomplete outcome data resulted in a „high risk' or a „low risk' of bias, e.g., missing data not reported for self-report measures, missing cases 80-95%

**Selective outcome reporting**<br>Reporting biases caused by the selective reporting of outcomes	
Low Risk	• The study protocol is available (and mentioned in the publication)<br>• The pre-defined primary/secondary outcomes were described as planned
High Risk	• The outcomes were not reported in accordance with the previously defined standards or not fully reported<br>• Primary outcomes that were not pre-specified in the study existed (except for outcomes with clear explanations, such as unexpected adverse effects)
Unclear	It is uncertain whether the selective outcome reporting resulted in a „high risk' or a „low risk' of bias, i.e., no study protocol is available

Supplementary Table 4

List of excluded studies with reasons for exclusion

Study ID	Title	Reason for exclusion
Abolhadi et al., 2022	Latent classes of post-traumatic stress disorder among survivors of the Bam Earthquake after 17 years.	Not focusing on prevalence
Acierno et al., 2009	A Pre-/Post-Disaster Epidemiological Study of Mental Health Functioning in Vietnam's Da Nang Province Following Typhoon Xangsane.	Sample duplicate (Amstadter 2009)
Aghamohammadi et al., 2021	Environmental heat-related health symptoms among community in a tropical city.	Not focusing on prevalence
Ahmad et al., 2010	Earthquake impact in a remote South Asian population: psychosocial factors and posttraumatic symptoms.	Not focusing on prevalence
Ahmer et al., 2006	Psychological morbidity among primary care attendees in earthquake affected areas of Northern Pakistan and Azad Kashmir	No validated instrument for specific mental disorder based on DSM/ICD
Alfuqaha et al., 2023	The Impact of Turkey and Syria Earthquakes on University Students: Posttraumatic Stress Disorder Symptoms, Meaning in Life, and Social Support	Sample not representative
Ali et al., 2023	Prevalence of Posttraumatic Stress Disorder and Depression Among Internally Displaced Persons in Mogadishu-Somalia.	Not focusing on a natural hazard
Amiri et al., 2022	Suicide attempts in residents of Bam: 17 years after the Bam earthquake in Iran	Less than 70% experienced natural hazard
Amstadter et al., 2010	Does Interviewer Status Matter? An examination of Lay Interviewers and Medical Doctor Interviewers in an Epidemiological Study in Vietnam.	Sample duplicate (Amstadter 2009)
Anwar et al., 2011	Reproductive health and access to healthcare facilities: risk factors for depression and anxiety in women with an earthquake experience.	No validated instrument for specific mental disorder based on DSM/ICD
Armenian et al., 2000	Loss as a determinant of PTSD in a cohort of adult survivors of the 1988 earthquake in Armenia: implications for policy.	No validated instrument for specific mental disorder based on DSM/ICD
Armenian et al., 2002	Risk factors for depression in the survivors of the 1988 earthquake in Armenia.	No validated instrument for specific mental disorder based on DSM/ICD
Ashok et al., 2019	Mental health status of flood-affected adults in rural Tamil Nadu: A cross-sectional study.	No validated instrument for specific mental disorder based on DSM/ICD
Asim et al., 2022	A survey of Post-Traumatic Stress Disorder, Anxiety and Depression among Flood Affected Populations in Kerala, India	Not focusing on prevalence
Aslam et al., 2010	Trauma, depression, anxiety, and stress among individuals living in earthquake affected and unaffected areas	No validated instrument for specific mental disorder based on DSM/ICD
Asnakew et al., 2019	Prevalence of post-traumatic stress disorder and associated factors among Koshe landslide survivors, Addis Ababa, Ethiopia: a community-based, cross-sectional study.	Not focusing on a natural hazard
Ataya et al., 2024	Sleep quality and mental health differences following Syria-Turkey earthquakes: A cross-sectional study	Sample not representative
Ayub et al., 2015	Rate and predictors of psychotic symptoms after Kashmir earthquake	Not focusing on prevalence
Banford Witting et al., 2020	The stigma of widowhood in war and disaster affected communities of Sri Lanka: Contextual paths between trauma exposure and mental health distress.	Not focusing on a natural hazard
Baniasadi et al., 2019	Bam Earthquake Survivors' Mental Health Status 12 Years after the Earthquake: A Population-Based Study.	No mental disorder according to ICD/DSM
Bastien, 2013	Empowering Communities from the Ground Up: Perspectives on a Lay Mental Health Project in Post-earthquake Haiti	Not focusing on prevalence
Berenz et al., 2013	Pretyphoon panic attack history moderates the relationship between degree of typhoon exposure and posttyphoon PTSD and depression in a Vietnamese sample.	Sample duplicate (Amstadter 2009)
Bertinelli et al., 2023	Earthquakes and mental health	Not focusing on prevalence
Bhandari et al., 2018	Psychological distress among the adult survivors of Kathmandu valley from Nepal's 2015 earthquake	Not focusing on prevalence
Blanc et al., 2020	Association between prenatal exposure to the Haiti 2010 earthquake, consequent maternal PTSD and autistic symptoms in offspring	Not focusing on prevalence

Study ID	Title	Reason for exclusion
Brown et al., 2013	Pre-typhoon socioeconomic status factors predict post-typhoon psychiatric symptoms in a Vietnamese sample. Social psychiatry and psychiatric epidemiology	Sample duplicate (Amstadter 2009)
Caldera et al., 2001	Psychological impact of the hurricane Mitch in Nicaragua in a one-year perspective.	No validated instrument for specific mental disorder based on DSM/ICD
Cénat et al., 2015	Psychometric properties of the Haitian Creole version of the Resilience Scale with a sample of adult survivors of the 2010 earthquake.	Sample duplicate (Cénat 2014)
Cénat et al., 2020	Sexual victimization, PTSD, depression, and social support among women survivors of the 2010 earthquake in Haiti: a moderated moderation model	Sample duplicate (Cénat 2014)
Chadda et al., 2007	Mental health problems following the 2005 earthquake in Kashmir: findings of community-run clinics.	No validated instrument for specific mental disorder based on DSM/ICD
Channaveerachari et al., 2015	Psychiatric and medical disorders in the after math of the uttarakhand disaster: assessment, approach, and future challenges.	No validated instrument for specific mental disorder based on DSM/ICD
Charak et al., 2014	Factor structure of PTSD, and relation with gender in trauma survivors from India	Sample duplicate (Charak 2014)
Chen et al., 2021	Trauma and well-being in colombian disaster contexts: effects of religious coping, forgivingness, and hope	Not focusing on prevalence
Chung et al., 2014	Posttraumatic stress disorder and psychiatric comorbidity following the 2010 flood in Pakistan: exposure characteristics, cognitive distortions, and emotional suppression.	Sample not representative
Chung et al., 2017	Posttraumatic stress symptoms, co-morbid psychiatric symptoms and distorted cognitions among flood victims of different ages.	Sample duplicate (Chung 2014)
Coêlho et al., 2004	Psychological Responses to Drought in Northeastern Brazil	No validated instrument for specific mental disorder based on DSM/ICD
Conrad et al., 2010	Examination of general health following Typhoon Xangsane: A pre-postanalysis	Sample duplicate (Amstadter 2009)
de la Fuente, 1986	Las consecuencias del desastre en la salud mental = Disaster consequences in regard to mental health	No validated instrument for specific mental disorder based on DSM/ICD
de la Fuente, 1990	The mental health consequences of the 1985 earthquakes in Mexico	No validated instrument for specific mental disorder based on DSM/ICD
Dasgupta & Basu, 2023	Mental health and socio-psychological manifestations of cyclone-induced water insecurity in the Indian Sundarban delta	No validated instrument for specific mental disorder based on DSM/ICD
Dell'Aringa et al., 2018	Rio's mountainous region ("Região Serrana") 2011 landslides: impact on public mental health system.	Not focusing on prevalence
Demirchyan et al., 2014	Rate and predictors of postpartum depression in a 22-year follow-up of a cohort of earthquake survivors in Armenia.	Sample duplicate (subsample Demirchyan 2022)
Demirchyan et al., 2015	Factor Structure and Psychometric Properties of the Posttraumatic Stress Disorder (PTSD) Checklist and DSM-5 PTSD Symptom Set in a Long-Term Postearthquake Cohort in Armenia	Sample duplicate (Demirchyan 2022)
Djarallah & Chorfi, 2012	The relationship between posttraumatic stress disorder and time perspective	Not focusing on prevalence
Do et al., 2019	Trauma Exposure and Mental Health Problems Among Adults in Central Vietnam: A Randomized Cross-Sectional Survey	Not focusing on a natural hazard
Eivazi et al., 2016	Comparative study of prevalence of post-traumatic stress disorder among survivors of bam earthquake 18 month after the event in bam and Kerman cities.	No mental disorder according to ICD/DSM
Escobar Carias et al., 2022	Flood disasters and health among the urban poor.	No validated instrument for specific mental disorder based on DSM/ICD
Exenberger et al., 2019	A cross-sectional study of mother-child agreement on PTSD symptoms in a south Indian post-tsunami sample	No validated instrument for specific mental disorder based on DSM/ICD
Fernando, 2008	Assessing mental health and psychosocial status in communities exposed to traumatic events: Sri Lanka as an example.	Not focusing on a natural hazard

Study ID	Title	Reason for exclusion
Frankenberg et al., 2008	Mental health in Sumatra after the tsunami	Not focusing on prevalence
Gargurevich et al., 2009	Factor structure of the Impact of Event Scale-Revised in two different Peruvian samples.	Not focusing on a natural hazard
George et al., 2012	Post-tsunami mental health: A cross-sectional survey of the predictors of common mental disorders in South India 9-11 months after the 2004 Tsunami.	No mental disorder according to ICD/DSM
Goenjian et al., 1994	Posttraumatic stress disorder in elderly and younger adults after the 1988 earthquake in Armenia.	Sample duplicate (Goenjian 1994)
Goenjian et al., 2008	Heritabilities of symptoms of posttraumatic stress disorder, anxiety, and depression in earthquake exposed Armenian families.	Not focusing on prevalence
Goenjian et al., 2015	Association of COMT and TPH-2 genes with DSM-5 based PTSD symptoms.	Not focusing on prevalence
Goenjian et al., 2018	Posttraumatic Stress Disorder 23 Years After the 1988 Spitak Earthquake in Armenia.	Sample duplicate (Khachadourian 2015)
Gunaratne et al., 2014	Trauma-related symptoms in Sri Lankan adult survivors after the tsunami: pretraumatic and peritraumatic factors.	Not focusing on prevalence
Hollifield et al., 2008	Symptoms and coping in Sri Lanka 20-21 months after the 2004 tsunami	N < 100
Hugelius et al., 2017	Health among disaster survivors and health professionals after the Haiyan Typhoon: a self-selected Internet-based web survey.	No mental disorder according to ICD/DSM
Isuru et al., 2019	Unconfirmed death as a predictor of psychological morbidity in family members of disappeared persons.	No validated instrument for specific mental disorder based on DSM/ICD
Iqbal & Sheikh, 2023	The devastating earthquake that struck Turkey and Syria: Post-traumatic stress disorder and mental health issues among the victims	Not focusing on prevalence
James et al., 2013	Lay mental health in the aftermath of disaster: Preliminary evaluation of an intervention for Haiti earthquake survivors	Sample not representative
Jang et al., 2020	Post-traumatic stress disorder and behavioral problems of parents and children after the 2015 Nepal earthquakes	No validated instrument for specific mental disorder based on DSM/ICD
Jones et al., 2011	Inequality, socioeconomic status, and social support in post-disaster mental health in Mexico	No validated instrument for specific mental disorder based on DSM/ICD
Juth et al., 2015	Post-Disaster Mental Health Among Parent-Child Dyads After a Major Earthquake in Indonesia.	Not focusing on prevalence
Kadri et al., 2006	Post-traumatic stress disorder in survivors of the Agadir earthquake (Morocco) in 1960.	N < 100
Kaniasty & Norris, 2008	Longitudinal linkages between perceived social support and posttraumatic stress symptoms: Sequential roles of social causation and social selection	Sample duplicate (Norris 2004)
Kar et al., 2004	Mental health consequences of the trauma of super-cyclone 1999 in orissa.	No validated instrument for specific mental disorder based on DSM/ICD
Kar et al., 2022	Anxiety, Depression, and Post-traumatic Stress a month after 2019 Cyclone Fani in Odisha, India	N < 100
Keraite et al., 2016	Exposure to conflict and disaster: A national survey on the prevalence of psychotic experiences in Sri Lanka.	No mental disorder according to ICD/DSM
Kesnold Mesidor, 2019	Posttraumatic growth in the 2010 Haitian earthquake survivors and its relationship with posttraumatic stress symptoms three years after the earthquake	No validated instrument for specific mental disorder based on DSM/ICD
Khachadourian et al., 2015	Loss and psychosocial factors as determinants of quality of life in a cohort of earthquake survivors.	Sample duplicate (Demirchyan 2022)
Khachadourian et al., 2016	A post-earthquake psychopathological investigation in Armenia: methodology, summary of findings, and follow-up.	Sample duplicate (Demirchyan 2022)
Khan et al., 2007	Frequency of post-traumatic stress disorder and its association with types of physical injuries and depression in earthquake victims.	No validated instrument for specific mental disorder based on DSM/ICD
Khatri et al., 2018	Effect of the 2015 Nepal Earthquakes on symptoms of common mental disorders among women who are pregnant.	No validated instrument for specific mental disorder based on DSM/ICD
Khatri et al., 2020	Experiences of an earthquake during pregnancy, antenatal mental health and infants' birthweight in Bhaktapur District, Nepal, 2015: a population-based cohort study.	No validated instrument for specific mental disorder based on DSM/ICD

Study ID	Title	Reason for exclusion
Kohn et al., 2005	Prevalence, risk factors and aging vulnerability for psychopathology following a natural hazard in a developing country.	Sample duplicate (Kohn 2005)
Koirala et al., 2020	Trauma related psychiatric disorders and their correlates in a clinical sample: A cross-sectional study in trauma affected patients visiting a psychiatric clinic in Nepal	Sample not representative
Kumar et al., 2023	Post-traumatic stress and depression following a landslide linked to the 2018 floods in Kerala, India: Relevance of screening.	N < 100
Kvestad et al., 2019	Earthquake Exposure and Post-traumatic Stress Among Nepalese Mothers After the 2015 Earthquakes.	No validated instrument for specific mental disorder based on DSM/ICD
Labarda & Chan, 2018	Sleep disturbances, posttraumatic stress, and psychological distress among survivors of the 2013 Super Typhoon Haiyan	Sample not representative
Lambert & Banford Witting, 2022	Mass trauma and long-term psychological distress: The role of economic deprivation among older widows in Sri Lanka	Not focusing on a natural hazard
Lavenda et al., 2017	Exploring DSM-5 criterion A in Acute Stress Disorder symptoms following natural hazard.	Not focusing on prevalence
Levy et al., 2009	Causal thinking after a tsunami wave: Karma beliefs, pessimistic explanatory style and health among Sri Lankan survivors	Not focusing on prevalence
Liang et al., 2022	Revisiting the factor structure of the insomnia severity index among survivors of the 2013 Super Typhoon Haiyan	Not focusing on prevalence
Lima et al., 1988	La atención primaria de salud mental en las víctimas del desastre de Armero, Colombia/ Mental health primary care among victims of the mudslide disaster in Armero, Colombia	No validated instrument for specific mental disorder based on DSM/ICD
Lima et al., 1990	Disasters and mental health: Experience in Colombia and Ecuador and its relevance for primary care in mental health in Latin America	No validated instrument for specific mental disorder based on DSM/ICD
Lima et al., 1991	Psychiatric disorders among poor victims following a major disaster: Armero, Colombia.	No validated instrument for specific mental disorder based on DSM/ICD
Lima & Pai, 1992	Response to the psychological consequences of disasters in Latin America	No validated instrument for specific mental disorder based on DSM/ICD
Lima et al., 1992	Psychiatric disorders among emotionally distressed disaster victims attending primary mental health clinics in Ecuador.	No validated instrument for specific mental disorder based on DSM/ICD
Lima et al., 1993	Emotional distress in disaster victims. A follow-up study.	No validated instrument for specific mental disorder based on DSM/ICD
Liu et al., 2022	DSM-5 Posttraumatic Stress Disorder Symptoms in Nonclinical Samples of Chinese and Pakistani Trauma-Exposed Adults Factor Structure and Invariance Across Culture	Not focusing on a natural hazard
Luce et al., 2022	Mental Health, Social Support, and Active Coping in Nepali Earthquake Survivors	No validated instrument for specific mental disorder based on DSM/ICD
Malhotra et al., 2010	Prevalence and correlates of clinically significant depressive symptoms among elderly people in Sri Lanka: Findings from a national survey	No validated instrument for specific mental disorder based on DSM/ICD
Martinez-Levy et al., 2022	Clinical, environmental, and molecular factors associated to the occurrence and persistence of posttraumatic stress disorder after an earthquake.	N < 100
Math et al., 2008	Comparative study of psychiatric morbidity among the displaced and non-displaced populations in the Andaman and Nicobar Islands following the tsunami.	No validated instrument for specific mental disorder based on DSM/ICD
Merrell, 2015	Dissociation differences between human-made trauma and natural hazard trauma	Not focusing on prevalence
Mesidor & Sly, 2019	Religious coping, general coping strategies, perceived social support, PTSD symptoms, resilience, and posttraumatic growth among survivors of the 2010 earthquake in Haiti	No validated instrument for specific mental disorder based on DSM/ICD
Minakshi et al., 2020	Mental Health Problems in Wake of Disaster: A Gendered Perspective	Not focusing on prevalence
Mohammadi & Ataei, 2009	Post-traumatic stress disorder in survivors of Bam earthquake	No validated instrument for specific mental disorder based on DSM/ICD

Study ID	Title	Reason for exclusion
Montazeri et al., 2005	Psychological distress among Bam earthquake survivors in Iran: a population-based study.	No mental disorder according to ICD/DSM
Mordeno & Cue, 2015	Examining the Latent Structure of Acute Stress Disorder Symptoms in Filipino-Victims of a Flashflood Disaster.	Less than a month after disaster
Mordeno et al., 2021	Development and validation of a DSM-5-based Generalized Anxiety Disorder Self-Report Scale: Investigating frequency and intensity rating differences	Not focusing on prevalence
Muir et al., 2019	Migration and Mental Health in the Aftermath of Disaster: Evidence from Mt. Merapi, Indonesia.	No validated instrument for specific mental disorder based on DSM/ICD
Nalipay & Mordeno, 2016	Emotional Intensity of Trauma Memory as Moderator of the Relationship Between Posttraumatic Cognitions and PTSD Symptoms	Sample duplicate (Mordeno 2017)
Nalipay & Mordeno, 2018	Positive metacognitions and meta-emotions as predictors of posttraumatic stress disorder and posttraumatic growth in survivors of a natural hazard	Not focusing on adults
Negi et al., 2018	Impact of a massive earthquake on adherence to antiretroviral therapy, mental health, and treatment failure among people living with HIV in Nepal.	Not focusing on prevalence
Nobakht et al., 2019	Risk factors of post-traumatic stress among survivors of the 2017 Iran earthquake: The importance of peritraumatic dissociation.	No validated instrument for specific mental disorder based on DSM/ICD
Noor, 2010	Profile and related factors of psychiatric problems among victims and survivors of Jogja earthquake.	Not focusing on prevalence
Norris et al., 2001	Postdisaster stress in the United States and Mexico: a cross-cultural test of the multicriterion conceptual model of posttraumatic stress disorder.	Not focusing on prevalence
Norris et al., 2002	Placing age differences in cultural context: A comparison of the effects of age on PTSD after disasters in the United States, Mexico, and Poland	Not focusing on prevalence
Norris et al., 2006	Early physical health consequences of disaster exposure and acute disaster-related PTSD	Sample duplicate (Norris et al., 2004)
Nzayisenga et al., 2022	Patterns of distress and psychosocial support 2 years post-displacement following a natural hazard in a lower middle income country.	No validated instrument for specific mental disorder based on DSM/ICD
Pan & Yang, 2021	PTSD symptoms, emotion regulation difficulties, and family functioning among trauma-exposed college students	Not focusing on prevalence
Pandit & Nakagawa, 2021	How Does Reciprocal Exchange of Social Support Alleviate Individuals' Depression in an Earthquake-Damaged Community?	Not focusing on prevalence
Patel et al., 2015	Posttraumatic stress disorders in adult victims of 2006 flood in Surat, Gujarat.	No validated instrument for specific mental disorder based on DSM/ICD
Patrick & Patrick, 1981	Cyclone '78 in Sri Lanka--the mental health trail.	No validated instrument for specific mental disorder based on DSM/ICD
Perera-Diltz et al., 2012	A cross-cultural exploration of posttraumatic stress disorder: Assessment, diagnosis, and recommended (Gestalt) treatment	Not focusing on prevalence
Pina Barba et al., 1991	Psychological effects on medical students caused by earthquakes of 1985.	No validated instrument for specific mental disorder based on DSM/ICD
Polat et al., 2023	Mental health in the aftermath of disasters	Not focusing on prevalence
Pomer et al., 2018	Relationships between psychosocial distress and diet during pregnancy and infant birthweight in a lower-middle income country: 'healthy mothers, healthy communities' study in Vanuatu.	No validated instrument for specific mental disorder based on DSM/ICD
Pyari & Ravindran, 2016	Sex differentials in the risk factors of post traumatic stress disorder among tsunami survivors in Tamil Nadu, India	No validated instrument for specific mental disorder based on DSM/ICD
Pyari et al., 2012	Risk factors of post-traumatic stress disorder in tsunami survivors of Kanyakumari District, Tamil Nadu, India.	No validated instrument for specific mental disorder based on DSM/ICD
Rafiey et al., 2017	Evaluating the Persian translation of the National Stressful Events Survey PTSD Short Scale in a sample of Iranian earthquake survivors	Not focusing on prevalence
Rafiey et al., 2018	Factor Structure of Persian Translation of the Patient Health Questionnaire in Iranian Earthquake Survivors	Not focusing on prevalence

Study ID	Title	Reason for exclusion
Rafiey et al., 2019	Prevalence and Determinants of PTSD 3 Years After an Earthquake in Iran.	Not focusing on prevalence
Rafiey et al., 2022	Exploring the buffering role of social capital in the development of posttraumatic stress symptoms among Iranian earthquake survivors.	Not focusing on prevalence
Rahill et al., 2022	Self-reported sleep disturbance patterns in urban haitians: A latent class analysis	No mental disorder according to ICD/DSM
Rahman et al., 2023	Impact of Disaster on mental health of women: A case study on 2022 flash flood in Bangladesh	No validated instrument for specific mental disorder based on DSM/ICD
Rajkumar et al., 2013	Lessons from the 2004 Asian tsunami: epidemiological and nosological debates in the diagnosis of post-traumatic stress disorder in non-Western post-disaster communities.	Sample duplicate (Rajkumar 2015)
Ranawaka & Dewaraja, 2006	Tsunami counselling project of the Sri Lanka National Institute of professional counsellors	No validated instrument for specific mental disorder based on DSM/ICD
Rescoe, 2020	Social capital and psychological stress in post-earthquake Haiti	No mental disorder according to ICD/DSM
Rezapour et al., 2023	Typologies of Posttraumatic Stress Disorder in the Bam Adult Population: 17 Years After Bam Earthquake	No validated instrument for specific mental disorder based on DSM/ICD
Riaz et al., 2015	Outcomes of belief in just world among victims of natural and man-made disaster: Moderating role of resilience	No validated instrument for specific mental disorder based on DSM/ICD
Roberson-Nay et al., 2013	Characteristics of individuals meeting criteria for new onset panic attacks following exposure to a typhoon.	Sample duplicate (Amstadter 2009)
Rose et al., 2021	Evaluating clinical outcomes of routinely delivered task-shared care for depression in rural Haiti.	Not focusing on prevalence
Samouei et al., 2021	Psychometric evaluation of a questionnaire for measuring factors related to mental health of women in an earthquake.	Not focusing on prevalence
Sattler et al., 2002	Hurricane Georges: A cross-national study examining preparedness, resource loss, and psychological distress in the US Virgin Islands, Puerto Rico, Dominican Republic, and the United States	Not focusing on prevalence
Sattler et al., 2006	El Salvador earthquakes: Relationships among acute stress disorder symptoms, depression, traumatic event exposure, and resource loss	Not focusing on prevalence
Sawangchai et al., 2023	Depression and suicidal ideation among Pakistani rural areas women during flood disaster.	Insufficient information on measures used
Seyedin et al., 2017	Psychological sequels of flood on residents of southeast Caspian region	No validated instrument for specific mental disorder based on DSM/ICD
Shabani et al., 2024	Prevalence and determinants of post-traumatic stress disorder five months after the 2019 huge flooding in Iran	No validated instrument for specific mental disorder based on DSM/ICD
Sonpaveerawong et al., 2019	Prevalence of Psychological Distress and Mental Health Problems among the Survivors in the Flash Floods and Landslide in Southern Thailand	No validated instrument for specific mental disorder based on DSM/ICD
Sriskandarajah et al., 2015	Predictors of violence against children in Tamil families in northern Sri Lanka.	Not focusing on a natural hazard
Stewart-Ibarra et al., 2017	Psychological Distress and Zika, Dengue and Chikungunya Symptoms Following the 2016 Earthquake in Bahia de Caraquez, Ecuador.	No validated instrument for specific mental disorder based on DSM/ICD
Suar & Khuntia, 2004	Caste, Education, Family and Stress Disorders in Orissa Supercyclone	No validated instrument for specific mental disorder based on DSM/ICD
Suar et al., 2002	Supercyclone in Orissa: An assessment of psychological status of survivors	No validated instrument for specific mental disorder based on DSM/ICD
Suar et al., 2007	Placing age differences in the context of the Orissa supercyclone: Who experiences psychological distress?	Sample duplicate (Suar 2002)
Suar et al., 2015	Bereavement, Postdisaster Trauma, and Behavioral Changes in Tsunami Survivors	No validated instrument for specific mental disorder based on DSM/ICD
Suar et al., 2017	Exposure, loss, and support predicting the dimensions of posttsunami trauma	No validated instrument for specific mental disorder based on DSM/ICD

Study ID	Title	Reason for exclusion
Suhail et al., 2009	Psychological health of earthquake survivors in Pakistan	Not focusing on prevalence
Tavernier et al., 2019	Sleep problems and religious coping as possible mediators of the association between tropical storm exposure and psychological functioning among emerging adults in Dominica	Not focusing on prevalence
Telles et al., 2009	Risk of posttraumatic stress disorder and depression in survivors of the floods in Bihar, India.	Not focusing on prevalence
Thomas et al., 2021	Exploring the psychiatric symptoms among people residing at flood affected areas of Kodagu district, Karnataka	Not focusing on prevalence
Thompson & Abel, 2009	Risk and protective factors in the development of post-traumatic stress disorder symptoms among a cohort of students in two tertiary institutions post Hurricane Ivan.	Not focusing on prevalence
Valladares-Garrido et al., 2023	Food insecurity in Piura, Peru, in the context of post-earthquake and the COVID-19 pandemic	Sample duplicate (Valladares-Garrido et al., 2022)
Viswanath et al., 2012	Psychological impact of the tsunami on elderly survivors.	No validated instrument for specific mental disorder based on DSM/ICD
Viswanath et al., 2013	Gender differences in the psychological impact of tsunami.	No validated instrument for specific mental disorder based on DSM/ICD
Viswanathan et al., 2019	Depression, Suicidal Ideation, and Resilience among Rural Farmers in a Drought-Affected Area of Trichy District, Tamil Nadu.	Not focusing on prevalence
Warsini et al., 2015	Psychometric evaluation of the Indonesian version of the Impact of Event Scale-Revised.	Not focusing on prevalence
Warsini et al., 2015	Posttraumatic stress disorder among survivors two years after the 2010 Mount Merapi volcano eruption: A survey study	No validated instrument for specific mental disorder based on DSM/ICD
Wickrama & Ketring, 2012	Change in the health of tsunami-exposed mothers three years after the natural hazard	No validated instrument for specific mental disorder based on DSM/ICD
Wickrama & Wickrama, 2008	Family context of mental health risk in Tsunami affected mothers: Findings from a pilot study in Sri Lanka	No validated instrument for specific mental disorder based on DSM/ICD
Wickrama & Wickrama, 2011	Perceived community participation in tsunami recovery efforts and the mental health of tsunami-affected mothers: findings from a study in rural Sri Lanka.	Sample duplicate (Wickrama 2008)
Wickrama et al., 2017	PTSD symptoms among tsunami exposed mothers in Sri Lanka: the role of disaster exposure, culturally specific coping strategies, and recovery efforts.	No validated instrument for specific mental disorder based on DSM/ICD
Woersching & Snyder, 2004	Earthquakes in El Salvador: a descriptive study of health concerns in a rural community and the clinical implications: Part III--Mental health and psychosocial effects.	No mental disorder according to ICD/DSM
Xiong et al., 2016	Prevalence of post traumatic stress disorder (PTSD) among flood victims in Malaysia: Difference between Kuala Lumpur and Kelantan	No validated instrument for specific mental disorder based on DSM/ICD
Yassini & Hosseini, 2006	Post traumatic stress disorders symptoms after bam earthquake.	No validated instrument for specific mental disorder based on DSM/ICD
Zahoor & Tahir, 2024	The Use of Telemedicine in Providing Mental Health Support for the Flood Victims of Pakistan	Not focusing on prevalence
Zhang et al., 2023a	Association of Optimism, Causal Thinking, and Karma Beliefs with PTSD and Depression 8 Years After the Tsunami in Sri Lanka	No validated instrument for specific mental disorder based on DSM/ICD
Zhang et al., 2023b	Heatwave and mental health	No validated instrument for specific mental disorder based on DSM/ICD

Supplementary Table 5

Severity of studied events according to the fatality-based Universal Disaster Severity Classification

Severity Class	Fatalities	Events
0	F < 1	2009 December floods (*k* = 1)
1	1 ≤ F ≤ 10	2021 Sullana earthquake (*k* = 1)
2	10 ≤ F ≤ 100	2017 Chiapas earthquake (*k* = 1)
3	100 ≤ F ≤ 1000	1991 Mount Pinatubo volcanic eruption (*k* = 1), 1997 hurricane Paulina (*k* = 1), 1999 Mexico floods (*k* = 2), 2006 typhoon Xangsane (*k* = 1), 2007 Pisco earthquake (*k* = 2), 2010 Ladakh floods (*k* = 1), 2014 Kashmir floods (*k* = 1), 2015 Chennai floods (*k* = 1), 2017 cyclone Mora (*k* = 1), 2017 El Niño Costero floods (*k* = 2), 2017 Puebla earthquake (*k* = 2), 2018 Kerala floods (*k* = 1), 2019 Bangladesh floods (*k* = 1)
4	1000 ≤ F ≤ 10000	2005 Zarand earthquake (*k* = 1), 2011 typhoon Washi (*k* = 1), 2013 typhoon Yolanda (*k* = 3), 2013 Uttarakhand floods (*k* = 1), 2015 Gorkha earthquake (*k* = 9)
5	10000 ≤ F ≤ 0.1M	1988 Spitak earthquake (*k* = 6), 1998 hurricane Mitch (*k* = 1), 2003 Bam earthquake (*k* = 6), 2005 Kashmir earthquake (*k* = 7), 2023 Turkey-Syria earthquake (*k* = 1)
6	0.1M ≤ F ≤ 1M	2004 Indian Ocean tsunami (*k* = 9), 2010 Haiti earthquake (*k* = 7)

Note. k indicates the number of studies investigating this event. As proposed by the authors, class 1 represents an emergency, classes 2 and 3 a disaster with significant damage, many serious injuries, and many fatalities, classes 4 and 5 a calamity with severe destruction, a major number of injuries, and great number of fatalities, and class 6 a catastrophe type 1 with widespread continental destruction, a massive number of injuries, and an extensive loss of life.

Caldera HJ, Wirasinghe SC (2022) A universal severity classification for natural disasters. *Natural Hazards* **111**(2), 1533-1573. doi:10.1007/s11069-021-05106-9.

Supplementary Table 6

Subgroup analyses on the influence of exposure to armed conflicts

Short-term

**outcome**	**conflict = yes**		**conflict = no**		***p*-value comparison**
	k	**prevalence in % (95% CI)**	***k***	**prevalence in % (95% CI)**	
PTSD	13	20.3 (12.2 – 61.7)	14	32.2 (20.7 – 46.3)	.150
depression	3	n.a.	7	23.2 (9.5 – 46.6)	n.a.
generalised anxiety disorder	2	n.a.	3	n.a.	

Long-term

**outcome**	conflict = yes		conflict = no		*p*-value comparison
	k	prevalence in % (95% CI)	*k*	prevalence in % (95% CI)	
PTSD	5	22.9 (10.7 – 42.4)	11	27.4 (16.5 – 42.0)	.673
depression	n.a. (only studies in settings without previous conflict)				
generalised anxiety disorder	n.a. (only short-term studies)				

Supplementary Table 7

Risk of bias assessment of included studies

Observational studies

Study ID	Selection of participants	Confounding variables	Outcome measure	Incomplete outcome data	Selective outcome reporting
**Abdollahi et al. (2011)**	high	low	high	unclear	unclear
**Acharya Pandey et al. (2023)**	high	high	high	low	unclear
**Adhikari Baral & K.C (2019)**	high	high	high	low	unclear
**Ali et al. (2012)**	high	high	high	low	unclear
**Amstadter et al. (2009)**	unclear	high	high	low	unclear
**Anwar et al. (2013)**	high	high	high	unclear	unclear
**Bailey et al. (2010)**	high	low	high	unclear	unclear
**Bandla et al. (2019)**	unclear	high	high	unclear	unclear
**Blanc et al. (2016)**	high	low	high	low	unclear
**Burnett Jr & Helm Jr (2013)**	high	low	high	high	unclear
**Cairo et al. (2010)**	moderate	high	high	unclear	unclear
**Cénat & Derivois (2014)**	high	high	high	low	unclear
**Cerdá et al. (2013)**	high	low	high	low	unclear
**Charak et al. (2014)**	high	high	high	low	unclear
**Cherian et al. (2020)**	high	low	high	low	unclear
**Contreras et al. (2018)**	high	high	high	unclear	unclear
**Dahal et al. (2018)**	high	high	high	unclear	unclear
**Dévieux et al. (2013)**	high	low	high	low	unclear
**Divsalar & Dehesh (2020)**	moderate	low	high	unclear	unclear
**Espinoza-Neyra et al. (2017)**	high	high	high	unclear	unclear
**Farhoudian et al. (2006)**	high	high	high	low	unclear
**Feder et al. (2013)**	high	high	high	low	unclear
**Flores et al. (2014)**	moderate	high	high	low	unclear
**Gay et al. (2020)**	unclear	unclear	low	low	unclear
**Ghaffari-Nejad et al. (2007)**	moderate	low	high	unclear	unclear
**Goenjian et al. (2021)**	high	low	high	low	unclear
**Goodarzi et al. (2011)**	high	low	high	unclear	unclear
**Hagh-Shenas et al. (2006)**	moderate	low	high	low	unclear
**Hashmi et al. (2011)**	high	high	high	low	unclear

**Study ID**	Selection of participants	Confounding variables	Outcome measure	Incomplete outcome data	Selective outcome reporting
**Horiguchi & Nakazawa (2021)**	high	high	high	low	unclear
**Howard et al. (1999)**	high	low	PTSD high<br>remaining low	low	unclear
**Jha et al. (2017)**	high	high	high	low	unclear
**Kabunga et al. (2022)**	low	high	high	low	unclear
**Kane et al. (2018)**	high	high	high	low	unclear
**Kar et al. (2014)**	high	low	high	unclear	unclear
**Khachadourian et al. (2015)**	high	low	high	unclear	unclear
**Kohn et al. (2005)**, **Kohn (2013)**	high	low	unclear	low	unclear
**Kumar et al. (2007)**	high	low	high	low	unclear
**Labarda et al. (2020)**	high	high	high	low	unclear
**Lommen et al. (2009)**	high	low	high	low	unclear
**Mamun et al. (2019)**	high	high	high	low	unclear
**Mamun et al. (2021)**	moderate	low	high	low	unclear
**Maya-Mondragón et al. (2019)**	high	high	high	unclear	unclear
**Miyazaki et al. (2006)**	unclear	unclear	high	unclear	unclear
**Mordeno & Hall (2017)**	high	unclear	high	low	unclear
**Mordeno et al. (2017)**	high	unclear	high	unclear	unclear
**Muldoon et al. (2017)**	high	high	high	unclear	unclear
**Naeem et al. (2011)**	moderate	high	high	low	unclear
**Niaz et al. (2006)**	high	high	high	unclear	unclear
**Norris et al. (2001)**	high	low	high	unclear	unclear
**Norris et al. (2004)**	high	low	high	low	unclear
**Pollack et al. (2016)**	moderate	high	high	low	unclear
**Rajkumar et al. (2015)**	high	low	high	low	unclear
**Rana et al. (2008)**	high	high	high	low	unclear
**Ranasinghe & Levy (2007)**	high	low	high	low	unclear
**Ranasinghe et al. (2023)**	high	low	high	low	unclear
**Reis et al. (2016)**	high	low	high	unclear	unclear
**Schwind et al. (2019)**	high	high	high	unclear	unclear
**Sharma et al. (2023)**	unclear	high	high	unclear	unclear

**Study ID**	Selection of participants	Confounding variables	Outcome measure	Incomplete outcome data	Selective outcome reporting
**Silvestre et al. (2014)**	high	low	high	unclear	unclear
**Soqia et al., (2023)**	high	high	high	unclear	unclear
**Srivastava et al. (2015)**	high	unclear	high	unclear	unclear
**Thapa et al. (2018)**	unclear	high	high	low	unclear
**Tiyuri et al. (2023)**	moderate	low	high	low	unclear
**Valladares-Garrido et al. (2022a, 2022b)**	high	unclear	high	unclear	unclear
**Wagenaar et al. (2012)**	high	low	high	unclear	unclear
**Wagle et al. (2021)**	high	high	high	low	unclear
**Wani et al., 2020**	high	high	high	unclear	unclear
**Zuñiga et al. (2019)**	high	unclear	high	unclear	unclear

Exposed vs. non-exposed comparisons

**Study ID**	**Selection of participants**	**Confounding variables**	**Outcome measure**	**Incomplete outcome data**	**Selective outcome reporting**
**Amiri et al., (2022)**	unclear	low	high	unclear	unclear
**Goenjian et al. (1994)**	high	low	high	unclear	unclear
**Najarian et al. (2017)**	unclear	low	high	unclear	unclear
**Perera-Diltz (2006)**	unclear	unclear	high	unclear	unclear
**Ramachandran et al. (2006)**	moderate	low	high	unclear	unclear
**Toukmanian et al. (2000)**	low	low	high	low	unclear

Supplementary Fig. 1

Prevalence estimates in specific subgroup

Study	Outcome	Sample	Cases	Total	Proportion	95%-CI
Abdollahi et al., 2011	PTSD	university sample	72	172	0.42	[0.34; 0.50]
Anwar et al., 2013	PTSD	married women of reproductive age	31	387	0.08	[0.06; 0.11]
Bailey et al., 2010	PTSD	family members of 12 families	70	200	0.35	[0.28; 0.42]
Bailey et al., 2010	Alcohol Dependence	family members of 12 families	9	200	0.04	[0.02; 0.08]
Burnett Jr & Helm Jr, 2013	PTSD	university sample	38	111	0.34	[0.25; 0.44]
Demirchyan et al., 2022	PTSD	employees of the Ministry of Health + relatives	102	719	0.14	[0.12; 0.17]
Dévieux et al., 2013	PTSD	HIV-positive individuals	52	104	0.50	[0.40; 0.60]
Horiguchi & Nakazawa, 2021	PTSD	mothers of young children	158	296	0.53	[0.48; 0.59]
Labarda et al., 2020	PTSD	socioeconomically disadvantaged households	44	345	0.13	[0.09; 0.17]
Mamun et al., 2019	Depression	women	72	111	0.65	[0.55; 0.74]
Maya-Mondragon et al., 2019	PTSD	waitlist from general health clinics	4916	44855	0.11	[0.11; 0.11]
Maya-Mondragon et al., 2019	Depression	waitlist from general health clinics	4123	44875	0.09	[0.09; 0.09]
Mordeno et al., 2017	PTSD	university sample	304	664	0.46	[0.42; 0.50]
Niaz et al., 2006	PTSD	university sample + displaced individuals	57	155	0.37	[0.29; 0.45]
Silvestre et al., 2014	PTSD	university sample	89	246	0.36	[0.30; 0.43]
Silvestre et al., 2014	Depression	university sample	78	246	0.32	[0.26; 0.38]
Wagle et al., 2021	PTSD	elderly	117	362	0.32	[0.28; 0.37]
Wagle et al., 2021	Depression	elderly	56	362	0.15	[0.12; 0.20]

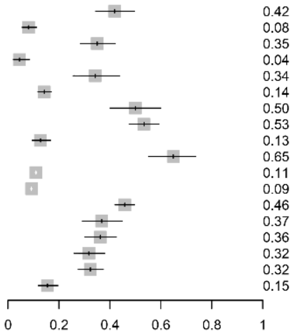

Supplementary Fig. 2

Plot for Leave-one-out analysis on PTSD short-term prevalence estimates

Study	Proportion	95%-CI	P-value	Tau2	Tau	I2
Omitting Adhikari Baral & K.C, 2019	0.26	[0.18; 0.37]	.	1.4360	1.1983	99%
Omitting Amstadter et al., 2009	0.28	[0.21; 0.38]	.	1.1599	1.0770	99%
Omitting Bandla et al., 2019	0.26	[0.18; 0.36]	.	1.4366	1.1986	99%
Omitting Cairo et al. 2010	0.26	[0.18; 0.37]	.	1.4366	1.1986	99%
Omitting Charak et al., 2014	0.26	[0.18; 0.37]	.	1.4359	1.1983	99%
Omitting Dahal et al., 2018	0.27	[0.19; 0.37]	.	1.4278	1.1949	99%
Omitting Espinoza-Neyra et al., 2017	0.26	[0.18; 0.35]	.	1.3981	1.1824	99%
Omitting Farhoudian et al., 2006	0.25	[0.18; 0.35]	.	1.3534	1.1634	99%
Omitting Hagh-Shenas et al., 2006	0.24	[0.17; 0.33]	.	1.1520	1.0733	99%
Omitting Jha et al., 2017 (sample 1)	0.27	[0.19; 0.37]	.	1.3641	1.1679	99%
Omitting Jha et al., 2017 (sample 2)	0.26	[0.18; 0.36]	.	1.4361	1.1984	99%
Omitting Kane et al., 2018	0.28	[0.20; 0.38]	.	1.2898	1.1357	99%
Omitting Kohn et al., 2005	0.27	[0.19; 0.37]	.	1.3841	1.1765	99%
Omitting Kumar et al., 2007	0.27	[0.19; 0.37]	.	1.4016	1.1839	99%
Omitting Miyazaki et al., 2006	0.26	[0.18; 0.36]	.	1.4235	1.1931	99%
Omitting Muldoon et al., 2017	0.25	[0.18; 0.35]	.	1.3744	1.1723	99%
Omitting Norris et al., 2001	0.26	[0.18; 0.36]	.	1.4364	1.1985	99%
Omitting Norris et al., 2004 (12 months)	0.27	[0.19; 0.37]	.	1.4265	1.1943	99%
Omitting Pollack et al., 2016	0.27	[0.19; 0.37]	.	1.3740	1.1722	99%
Omitting Ramachandran et al., 2006	0.25	[0.18; 0.35]	.	1.3424	1.1586	99%
Omitting Schwind et al., 2019	0.27	[0.19; 0.37]	.	1.3657	1.1686	99%
Omitting Soqia et al., 2023	0.26	[0.18; 0.35]	.	1.3877	1.1780	99%
Omitting Srivastava et al., 2015	0.25	[0.17; 0.34]	.	1.2781	1.1305	99%
Omitting Valladares-Garrido et al., 2022a	0.27	[0.18; 0.37]	.	1.4309	1.1962	99%
Omitting Wani et al., 2020	0.26	[0.18; 0.37]	.	1.4367	1.1986	99%
Omitting Zuñiga et al., 2019	0.26	[0.18; 0.36]	.	1.4310	1.1963	99%
**Random effects model**	**0.26**	**[0.18; 0.36]**	**.**	**1.3798**	**1.1747**	**99%**

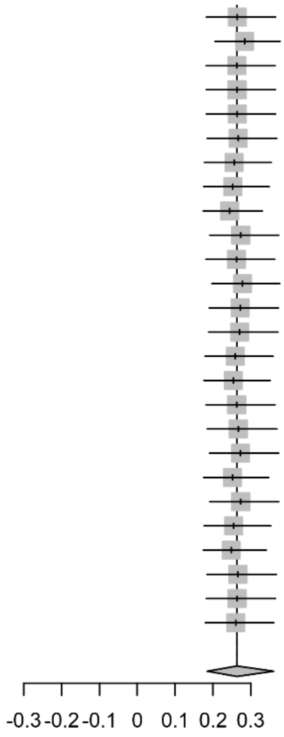

Supplementary Fig. 3

Plot for Leave-one-out analysis on PTSD longt-term prevalence estimates

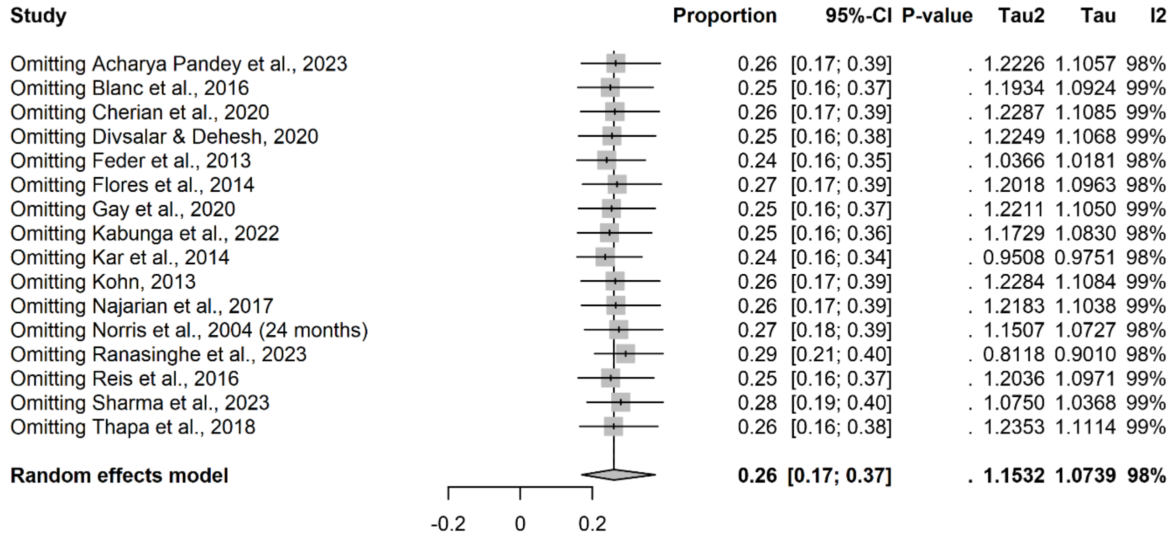

Supplementary Fig. 4

Plot for Leave-one-out analysis on depression short-term prevalence estimates

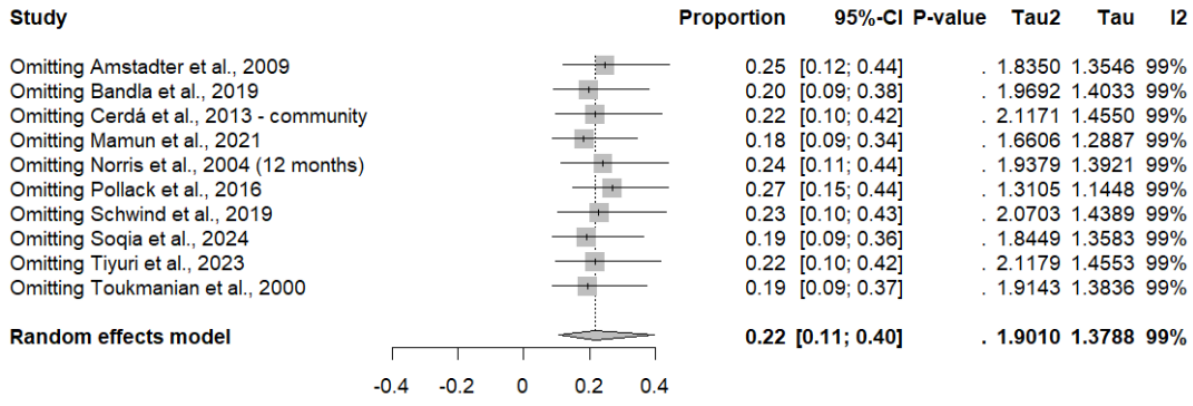

Supplementary Fig. 5

Plot for Leave-one-out analysis on depression long-term prevalence estimates

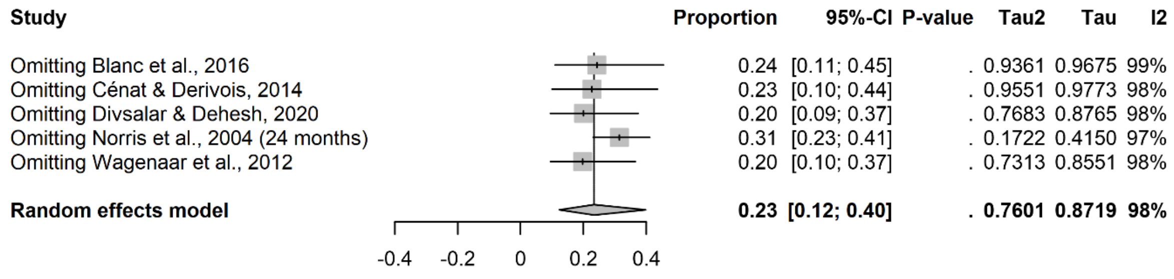

Supplementary Fig. 6

Plot for Leave-one-out analysis on GAD short-term prevalence estimates

Study	Proportion	95%-CI	P-value	Tau2	Tau	I2
Omitting Amstadter et al., 2009	0.24	[0.09; 0.52]	.	1.5317	1.2376	99%
Omitting Mamun et al., 2021	0.12	[0.03; 0.37]	.	2.2743	1.5081	100%
Omitting Pollack et al., 2016	0.21	[0.06; 0.54]	.	2.2935	1.5144	99%
Omitting Soqia et al., 2023	0.11	[0.03; 0.32]	.	1.9117	1.3826	99%
Omitting Valladares-Garrido et al., 2022b	0.15	[0.03; 0.49]	.	2.8945	1.7013	99%
**Random effects model**	**0.16**	**[0.05; 0.42]**	**.**	**2.3269**	**1.5254**	**99%**

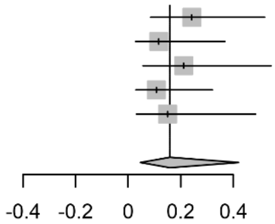

Supplement: Kip et al. supplementary material [file S2045796024000672sup001.pdf]
